# Tiered Clinician Vaccine Communication Strategy to Improve Childhood Vaccine Uptake: A Cluster Randomized Clinical Trial

**DOI:** 10.1001/jamanetworkopen.2025.7814

**Published:** 2025-04-30

**Authors:** Douglas J. Opel, Jeffrey D. Robinson, Chuan Zhou, Kathryn Colborn, Heather Spielvogle, Anna Furniss, Christine Spina, Cathryn Perreira, Sean T. O’Leary

**Affiliations:** 1 Department of Pediatrics, University of Washington School of Medicine, Seattle; 2 Seattle Children’s Research Institute, Seattle, Washington; 3 Department of Communication, Portland State University, Portland, Oregon; 4 Adult and Child Center for Outcomes Research and Delivery Science, University of Colorado School of Medicine and Children’s Hospital Colorado, Aurora; 5 Department of Medicine, University of Colorado Anschutz Medical Campus, Aurora; 6 Department of Biostatistics and Informatics, Colorado School of Public Health, Aurora; 7 Department of Pediatrics, University of Colorado Anschutz Medical Campus, Aurora

## Abstract

**Importance:**

No experimental studies to date have tested the effectiveness of clinician communication strategies to improve vaccine uptake among children of parents with negative vaccine attitudes.

**Objective:**

To evaluate the effect of a tiered vaccine communication strategy on children of parents with negative vaccine attitudes.

**Design, Setting, and Participants:**

This cluster randomized clinical trial was conducted from September 1, 2019, to March 31, 2023, at primary care pediatric clinics in Colorado (n = 12) and Washington State (n = 12). Parents with negative vaccine attitudes who had infants 2 months or younger and were receiving health supervision at a participating clinic during the intervention period were eligible for the study.

**Intervention:**

Tiered strategy in which clinicians initiated vaccine discussions with all parents using a presumptive format and then used motivational interviewing with parents who verbally resisted recommended vaccines. Clinicians at intervention clinics received training on the tiered vaccination strategy, whereas clinicians at control clinics practiced usual care.

**Main Outcomes and Measures:**

Child vaccination status at 19 months of age characterized as percentage of days underimmunized (DU) in receiving recommended doses of 8 routinely recommended vaccines, with 0% DU described as up to date (UTD) on time.

**Results:**

A total of 937 parent-child dyads (intervention: n = 586; control: n = 351) were included in analysis; 434 (46.3%) had an annual household income greater than $75 000 and 682 (72.8%) had some college, a 2-year degree, or more education. In bivariable analyses, more intervention (vs control) participants were UTD on time (130 [22.2%] vs 64 [18.2%]), but intervention (vs control) participants had higher mean (SD) percentage of DU (32.1% [37.9%] vs 23.7% [32.4%]). Variation was observed across states, with intervention participants in Washington (vs Colorado) having a lower mean (SD) percentage of DU (22.4% [31.8%] vs 39.6% [40.1%]) and more infants who were UTD on time (77 [30.0%] vs 53 [16.1%]). In multivariable regression models, there was no difference between intervention and control arms in mean (SD) percentage of DU (adjusted incidence rate ratio, 1.06; 95% CI, 0.69-1.63) or UTD on time (adjusted odds ratio, 1.45; 95% CI, 0.69-3.04), although there were increased odds of being UTD on time among Washington intervention (vs control) participants (adjusted odds ratio, 2.47; 95% CI, 1.09-5.59).

**Conclusions and Relevance:**

In this cluster randomized clinical trial of a tiered vaccine communication strategy, vaccine uptake of children of parents with negative vaccine attitudes did not improve, although state-specific effects were observed.

**Trial Registration:**

ClinicalTrials.gov ID: NCT03885232

## Key Points

**Question:**

What is the effect of a clinician vaccine communication strategy designed to promote vaccine uptake on the vaccination status of children of parents with negative vaccine attitudes?

**Findings:**

In this cluster randomized clinical trial involving 937 parent-child dyads at 24 clinics in Washington and Colorado, no significant overall difference was found in percentage of days underimmunized among children at intervention and control clinics, although intervention (vs control) children in Washington had significantly increased odds of receiving all vaccinations on time through 19 months of age.

**Meaning:**

The communication strategy did not improve vaccine uptake among children of parents with negative vaccine attitudes, although state-specific effects were observed.

## Teaser

This cluster randomized clinical trial of parent-child dyads examines the effect of a clinician vaccine communication strategy on vaccine uptake among children of parents with negative vaccine attitudes.

## Introduction

Overall vaccination coverage of children in the US remains high.^1^ However, since the COVID-19 pandemic, childhood vaccination rates have decreased, with persistent disparities by race and ethnicity, insurance status, and geographic area.^2^ Additionally, compared with prepandemic levels, a significantly higher proportion of parents are concerned about the safety and adverse effects of routine childhood vaccines,^3^ and more parents are distrustful of vaccine information.^4^ By 2022, vaccine hesitancy among specific groups of parents had significantly increased,^5^ and by the 2023 to 2024 school year, the proportion of parents who chose to opt their child out of required vaccines had increased to its highest level ever.^6^

Pediatric clinicians are critical to sustaining childhood vaccine coverage rates. Notably, pediatric clinicians are the most common source of vaccine information for parents^7^ and are the most trusted source for vaccine safety information,^8^ a trust that persisted during the COVID-19 pandemic.^9^ For these reasons, recent research has focused on clinician communication behaviors that positively influence parents’ behaviors toward childhood vaccines. In observational studies, use of a presumptive format (eg, “Sara is due for 2 shots today.”), compared with a participatory one (eg, “How do you feel about vaccines today?”), was associated with fewer parents voicing resistance to recommended childhood vaccines^10^ and increased odds that parents accepted all recommended vaccines by the visit’s end.^11^ In addition, in a study using an experimental design, motivational interviewing (MI) increased adolescent vaccine uptake.^12^

Two major gaps exist in our understanding of how to effectively communicate with parents about early childhood vaccines. First, although randomized clinical trials of clinician vaccine communication strategies have been published in the adolescent context,^12,13,14^ to our knowledge, only 1 randomized trial of a clinician communication strategy exists in the childhood context and had negative results.^15^ Other experimental work^16^ of face-to-face interventions with parents has largely involved delivering vaccine information, and the quality of this work and the risk of bias confound drawing definitive conclusions. Second, existing experimental work^17^ that measures the effect on vaccine uptake only assesses intervention effects on all enrolled parents, not specifically on parents with negative vaccine attitudes, arguably the most important population for targeted interventions.

We present the results of a cluster randomized clinical trial (cRCT) in which we tested the effect of a clinician vaccine communication strategy on vaccine uptake among children of parents with negative vaccine attitudes. The communication strategy tested incorporated use of the presumptive format and MI. We hypothesized that children of parents exposed to the strategy would have higher vaccine uptake than children of parents who were not exposed.

## Methods

### Study Overview

We conducted a cRCT in pediatric primary care clinics in Colorado and Washington State from September 1, 2019, to March 31, 2023. The protocol has been previously published.^18^ This study, Presumptively Initiating Vaccines and Optimizing Talk with Motivational Interviewing (PIVOT with MI) Trial, was approved by the Colorado Multiple Institutional Review Board, the Washington State Institutional Review Board, and the Swedish Health Services Institutional Review Board. Parents consented to participate with completion of an enrollment survey that contained information about the study and its risks and benefits. The Consolidated Standards of Reporting Trials Extension (CONSORT Extension) reporting guideline for cRCTs was followed. The trial protocol can be found in Supplement 1.

### Study Design and Setting

This study was a 2-arm cRCT. Study arms included a control arm, in which clinicians practiced usual care, and an intervention arm, in which clinicians used a tiered vaccine communication strategy. Clinics were recruited through 2 regional practice-based research networks in each state and through outreach efforts to individual clinics by the principal investigators (D.J.O. and S.T.O.). We considered a clinic enrolled when clinic leadership agreed to participate.

### Participants

All English- and Spanish-speaking parents with an infant 2 months or younger receiving health supervision at a participating clinic during the intervention period were eligible. For parents of twins, the first listed child was considered eligible. Nontwin siblings of enrolled children who were born to the same parent during the study period were also considered ineligible if they were clearly identifiable via the parent’s name and address entered on the enrollment survey.

Our primary study population was children of parents with negative vaccine attitudes. We identified these children by administering the short form of the validated Parent Attitudes about Childhood Vaccines^19,20,21^ (PACV-SF) to all eligible parents at check-in for their infant’s health supervision visit (eTable 1 in Supplement 2). The PACV-SF is scored on a scale of 0 to 4, with higher scores indicating more negative vaccine attitudes; parents with negative vaccine attitudes were defined as those who scored 2 or higher.^22^

### Randomization and Blinding

Clinics were the unit of randomization. Enrolled clinics within each state were randomly assigned by study analysts to a study arm using covariate-constrained randomization to ensure balanced arms on key clinic-level variables that may influence study outcomes. We chose this method given the potential of covariate imbalance in cRCTs with relatively few clusters.^23^ We selected 3 variables for covariate-constrained randomization: the proportion of parents with negative vaccine attitudes, the number of clinicians, and the proportion of Vaccines for Children–eligible patients. We determined the proportion of parents with negative vaccine attitudes at each enrolled clinic by administering the PACV-SF to all English- and Spanish-speaking parents on check-in for their infant’s first visit for a period of 2 months before the start of the intervention period. We determined the number of clinicians and the proportion of Vaccines for Children–eligible patients through a survey administered to each clinic manager or lead.

Given the need to train clinicians at intervention clinics on the vaccine communication strategy, it was not possible to blind clinicians or investigators to study arm allocation. However, study analysts were blinded to study arm allocation through analysis. We minimized selection bias by distributing the PACV-SF to all parents with children 2 months or younger who received health supervision at participating clinics during the intervention period. We blinded clinicians to parent PACV-SF scores to minimize participant bias and embedded the PACV-SF in a larger survey to minimize parent ascertainment bias.

### Intervention

The study intervention was a tiered clinician vaccine communication strategy in which clinicians were trained to use a presumptive format to initiate the childhood vaccine discussion with all parents at visits in which a child was eligible to receive 1 or more vaccine doses followed by use of MI if parents verbally resisted the recommended vaccines. This strategy therefore combined 2 evidence-based vaccine communication strategies.^10,11,12^

Clinicians (physicians, advanced registered nurse practitioners, and certified physician assistants) at intervention clinics completed a training curriculum on the communication strategy, which included a web module, an in-person baseline training, and 2 in-person or virtual refresher trainings. Description of the curriculum and rates of participation (eAppendix in Supplement 2) have been published.^24^ Other clinicians (eg, nurses and medical assistants) and office staff were invited to attend in-person trainings.

### Data Collection

Parents completed the PACV-SF at enrollment. The PACV-SF was embedded within a larger survey about other infant care topics (ie, car seat safety, feeding, and infant sleep and crying). This survey was included in the clinic’s standard intake paperwork that parents completed before the visit and offered a disclaimer stating completion was voluntary and for research purposes only. This survey also included demographic items (ie, parent age, child birth order, household income, marital status, parent self-designated race and ethnicity, relationship to child, and number of children in their household) and, for parent participants in Washington State, authorization to access their child’s vaccine data in the Washington State Immunization Information System (WA IIS).

The survey also asked parents for permission to contact them for future studies if interested. For enrolled parents with negative vaccine attitudes at intervention clinics who provided permission, study staff contacted them via phone and/or email to assess their willingness to have one of their child’s future health supervision visits through 18 months of age videotaped. Written informed consent was obtained from intervention parents and clinicians before videotaping, and both were given the right to review the videotape after the visit and delete any or all portions. Redacted transcripts of the vaccine discussion from videotaped encounters were used in refresher trainings.

We obtained immunization data from the Colorado Immunization Information System (CIIS) for children at Colorado clinics and from the WA IIS for children at Washington clinics whose parents provided authorization. We extracted CIIS immunization data using 25 CVX codes and WA IIS immunization data using 26 specific WA IIS numeric codes (eTable 2 in Supplement 2) that corresponded to the 8 vaccines of interest recommended from birth to 19 months of age (ie, hepatitis B, rotavirus, diphtheria and tetanus toxoids and acellular pertussis, *Haemophilus influenzae* type B, pneumococcal conjugate, inactivated polio virus, measles-mumps-rubella, and varicella).

For children at Washington clinics whose parents did not provide authorization for use of WA IIS or for whom a match was not found in WA IIS, we obtained immunization data from their child’s clinic medical records. Immunization data from clinic medical records were also used for children at Colorado clinics who could not be matched in CIIS. Children with no CIIS or WA IIS record but at least 1 clinic visit were included in analysis even if there was indication from clinic staff that the child changed clinics or moved out of state. Children who had no CIIS or WA IIS record or no record of a clinic visit were presumed to have incorrect demographic data (eg, name and date of birth) and excluded from analysis.

### Outcomes

Our primary outcome was child immunization status at 19 months of age, characterized as percentage of days underimmunized (DU) for all doses of the 8 vaccines of interest recommended in this age range, with 0% DU described as up to date (UTD) on time. To calculate DU, we used previously established methods^19,20,25^ based on the recommended ages and intervals between doses provided by the Advisory Committee on Immunization Practices^26^ for summing the days late for each dose of the 8 vaccines (eTable 3 in Supplement 2). There was a maximum of 23 recommended doses, although this number varied depending on the brand of vaccine received. If a child received no vaccines, the total maximum DU was a sum of the total possible days late for each dose through 19 months of age (2830 days). Percentage of DU was calculated by dividing DU by the total maximum DU. Children who received all doses of the 8 vaccines within the accepted age range (converted to days) for each dose, accounting for the minimum acceptable age and interval for each dose, were considered to have 0% DU.

### Sample Size

On the basis of preliminary data that children of parents with negative attitudes about vaccines had a mean (SD) percentage of DU of 26.0% (29.8%),^19^ we estimated that enrollment of 600 children of parents with negative vaccine attitudes (300 per arm and 25 at each of the 24 study clinics), assuming an α = .05, an SD of 20, and an intraclass correlation coefficient for within-clinic correlation of 0.02,^15^ would detect with adequate power (≥90%) a decrease of 7 percentage points in DU. Assuming a 10% prevalence of parents with negative vaccine attitudes,^15^ we planned to approach 6000 total parent-child dyads to reach our sample size goal.

### Statistical Analysis

We conducted an intention-to-treat analysis with the parent-child dyad as the unit of analysis. All analyses were preplanned except where noted. We summarized the mean percentage of DU and the proportion who were UTD on time using descriptive statistics by study arm overall and by state. We also examined baseline characteristics of parents by study arm using descriptive statistics to assess for any unbalanced confounders to include in regression models. We included race and ethnicity in this examination because of their association with immunization status.

We used generalized linear mixed-effects regression models to examine the effect of the intervention on our primary outcome: for mean percentage of DU, we applied a mixed zero-inflated β-regression model, and for UTD on time, we applied a mixed logistic regression model. All models included fixed binary factors for treatment arm and state as well as a random effect for clinic to account for correlation within clinics. We also conducted a post hoc analysis of the effect of the intervention on our primary outcome by state. These models were similar to the aforementioned models but excluded a fixed binary factor for state. Fully adjusted models also included unbalanced parent demographics across study arms. For all inferences, a 2-sided *P* < .05 was considered statistically significant.

## Results

We enrolled 12 clinics from Washington and 12 from Colorado. Within each state, we randomized 6 clinics to the intervention and control arms. There was a balanced distribution of the variables used in the covariate-constrained randomization across arms (eTable 4 in Supplement 2).

**Figure 1.**
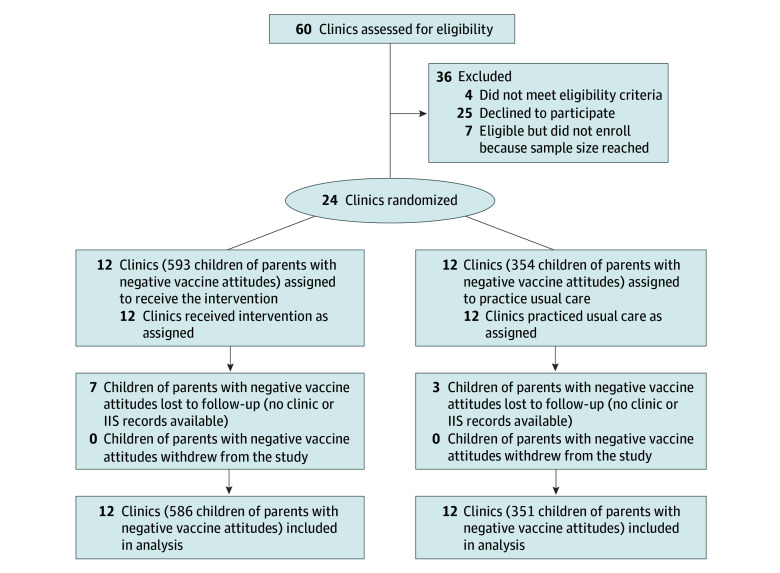
CONSORT Diagram IIS indicates Immunization Information System.

We enrolled 947 children of parents with negative vaccine attitudes (593 from intervention clinics and 354 from control clinics), with 937 parent-child dyads (intervention: n = 586; control: n = 351) included in analyses (Figure 1). The 10 children excluded had no CIIS or WA IIS record or record of a clinic visit. Twenty-one patients (2.2%) were American Indian or Alaska Native, 58 (6.2%) were Asian, 41 (4.4%) were Black or African American, 9 (1.0%) were Native Hawaiian or Pacific Islander, 670 (71.5%) were White, 59 (6.3%) were more than 1 race, and 79 (8.4%) had missing race data. A total of 434 parents (46.3%) had an annual household income greater than $75 000, and 682 (72.8%) had some college, a 2-year degree, or more education. (Table 1). Overall, the mean (SD) percentage of DU was 28.9% (36.1%), with an intraclass correlation coefficient for within-clinic correlation of 0.09 (95% CI, 0.05-0.28). A total of 194 parents (20.7%) were UTD on time.

**Table 1.** Demographics of Parent Participants Across Arms

Characteristic	No. (%) of parents	
	Control (n = 351)	Intervention (n = 586)
Parent age, y		
18-29	130 (37.0)	230 (39.2)
≥30	216 (61.5)	330 (56.3)
Missing	5 (1.4)	26 (4.4)
Parent’s marital status		
Single, separated, widowed, or divorced	35 (10.0)	65 (11.1)
Married or living with a partner	310 (88.3)	496 (84.6)
Missing	6 (1.7)	25 (4.3)
Parental educational level		
High school graduate or GED or less	65 (18.5)	155 (26.4)
Some college, 2-y degree, or more	278 (79.2)	404 (68.9)
Missing	8 (2.3)	27 (4.6)
Annual household income, $		
≤75 000	136 (38.7)	289 (49.3)
>75 000	191 (54.4)	243 (41.5)
Missing	24 (6.8)	54 (9.2)
Relationship to child		
Mother	309 (88.0)	491 (83.8)
Father	37 (10.5)	71 (12.1)
Missing	5 (1.4)	24 (4.1)
No. of children		
1	204 (58.1)	336 (57.3)
≥2	139 (39.6)	223 (38.1)
Missing	8 (2.3)	27 (4.6)
Parent ethnicity		
Hispanic or Latino	69 (19.7)	96 (16.4)
Not Hispanic or Latino	269 (76.6)	448 (76.4)
Missing	13 (3.7)	42 (7.2)
Parent race		
American Indian or Alaska Native	7 (2.0)	14 (2.4)
Asian	35 (10.0)	23 (3.9)
Black or African American	22 (6.3)	19 (3.2)
Native Hawaiian or Pacific Islander	5 (1.4)	4 (0.7)
White	238 (67.8)	432 (73.7)
>1 Race	20 (5.7)	39 (6.7)
Missing	24 (6.8)	55 (9.4)
Parent PACV-SF score		
2	211 (60.1)	323 (55.1)
3	101 (28.8)	182 (31.1)
4	39 (11.1)	81 (13.8)

Abbreviations: GED, General Educational Development; PACV-SF, Parent Attitudes about Childhood Vaccines (short form).

**Figure 2.**
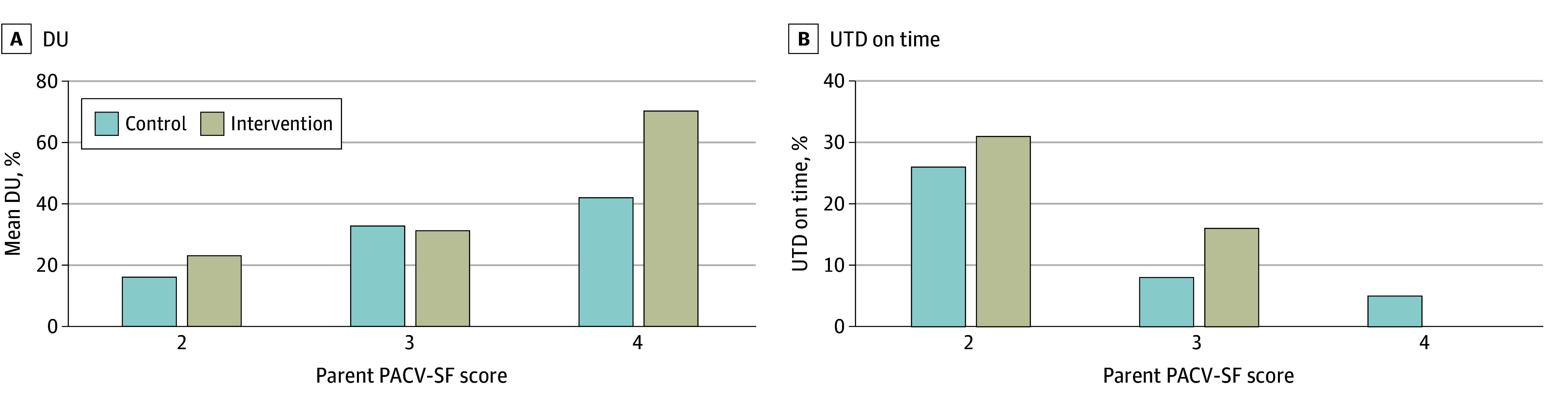
Mean Percentage of Days Underimmunized (DU) and Up to Date (UTD) on Time by Parent Attitudes About Childhood Vaccines (PACV-SF) Score Higher PACV-SF scores indicate more negative vaccine attitudes.

In bivariable analyses, the mean percentage of DU as well as proportion UTD on time across study arms is given in Table 2. Intervention (vs control) participants had a higher mean (SD) percentage of DU (32.1% [37.9%] vs 23.7% [32.4%]), but more were UTD on time (130 [22.2%] vs 64 [18.2%]), with different effects observed by state: intervention participants in Washington (vs Colorado) having a lower mean (SD) percentage of DU (22.4% [31.8%] vs 39.6% [40.1%]) and more UTD on time (77 [30.0%] vs 53 [16.1%]). We also observed increasing mean (SD) percentage of DU and decreasing proportions of UTD on time with increasing PACV-SF score tier (Figure 2).

**Table 2.** Mean Percentage of DU and Proportion With UTD on Time Across Study Groups Overall and Across States

Variable	Overall		Washington		Colorado	
	Control (n = 351)	Intervention (n = 586)	Control (n = 165)	Intervention (n = 257)	Control (n = 186)	Intervention (n = 329)
DU, mean (SD), %	23.7 (32.4)	32.1 (37.9)	27.5 (33.4)	22.4 (31.8)	10.3 (31.1)	39.6 (40.1)
UTD on time, No. (%)^a^	64 (18.2)	130 (22.2)	28 (17.0)	77 (30.0)	36 (19.4)	53 (16.1)

Abbreviations: DU, days underimmunized; UTD, up to date.

^a^ UTD on time is 0% DU.

In multivariable regression models, no significant difference was found in mean (SD) percentage of DU (adjusted incidence rate ratio, 1.06; 95% CI, 0.69-1.63) or UTD on time (adjusted odds ratio, 1.45; 95% CI, 0.69-3.04) between the intervention and control arms (Table 3). There was also no significant difference in the mean (SD) percentage of DU within each state. However, there was an increased odds of being UTD on time among Washington intervention (vs control) participants (adjusted odds ratio, 2.47; 95% CI, 1.09-5.59).

**Table 3.** Effect of Intervention on Outcomes Using Mixed-Effect Models

	IRR or OR (95% CI)^a^	Adjusted IRR or adjusted OR (95% CI)^b^
**Mean percentage DU**		
Overall	1.05 (0.70-1.58)	1.06 (0.69-1.63)
Washington	1.02 (0.75-1.39)	0.98 (0.70-1.38)
Colorado	1.06 (0.54-2.10)	1.14 (0.56-2.32)
**UTD on time (0% DU)**		
Overall	1.47 (0.71-3.04)	1.45 (0.69-3.04)
Washington	2.53 (1.14-5.61)	2.47 (1.09-5.59)
Colorado	0.81 (0.26-2.52)	0.79 (0.24-2.57)

Abbreviations: DU, days underimmunized; IRR, incidence rate ratio; OR, odds ratio; UTD, up to date.

^a^ IRRs are given for mean percentage of DU. IRRs were estimated using a zero-inflated β-regression model; all models accounted for clustering within clinics and for the overall sample were adjusted for state. ORs are given for UTD on time. ORs were estimated using mixed logistic regression; all models accounted for clustering within clinics and for the overall sample were adjusted for state.

^b^ These models were also adjusted for unbalanced demographic variables (for the overall sample, these were educational level, income, and race; for the Washington and Colorado study samples, these were educational level and income; race was excluded because of small cell sizes.

## Discussion

Overall, we found that the PIVOT with MI clinician vaccine communication strategy did not improve vaccine uptake among children of parents with negative vaccine attitudes. This is an important finding given that several previous observational and experimental studies found that use of the presumptive format or MI was effective,^10,11,12,13,27,28^ an evidence base that has supported inclusion of these communication strategies in current guidelines.^29^ There are several possible reasons for this discordance.

First, we studied the effect of a combined communication strategy, whereas previous studies^10,11,13,27,28^ primarily studied the effect of the presumptive format or MI alone. It is possible that these strategies used in isolation are effective but in combination are ineffective. It is notable, however, that one cRCT combined training on MI with use of the presumptive format and had a positive effect on HPV uptake.^12^

Second, we focused on the effect of our intervention on uptake of several childhood vaccines, whereas previous experimental studies tested the effect of these communication strategies on adolescent vaccine uptake and primarily 1 vaccine: human papillomavirus vaccine.^12,13^ Not only might there be a positive effect of these communication strategies on a single vaccine rather than a group of vaccines, but also the childhood and adolescent communication contexts may be sufficiently different to yield different effects. The previous experimental study^15^ testing the effect of a different vaccine communication intervention in the childhood vaccine context also had negative results. To our knowledge, there are no experimental studies to date that have tested either the presumptive format or MI strategies alone in the childhood vaccine context.

Third, our study is the first experimental study, to our knowledge, of a face-to-face communication intervention specifically designed to improve vaccination status in children of parents with negative vaccine attitudes. Because previous experimental studies have calculated the effect of a communication intervention among all parents, results from these studies mask the intervention’s effects among parents with negative vaccine attitudes. However, as we observed, an intervention’s desired effect may be blunted as a parent’s level of negative vaccine attitudes increases. It is possible that the positive effects observed by previous studies^13,14^ are attributable to an intervention’s effect on parents without negative attitudes because these parents predominate.

We observed a significant improvement in the proportion of children in the intervention (vs control) arm who were UTD on time in Washington, but not Colorado, despite there being no significant difference in the mean percentage of DU between intervention and control participants in either state. The reason for this differential effect by state is unclear. Our training protocol was standardized and delivered similarly across all clinics in both states. It is possible that more clinicians in intervention clinics in Colorado (vs Washington) did not use or implement the strategy as intended. There are also numerous personal, social, and local environmental factors that influence parental vaccine decision-making beyond clinician communication, and it is possible that these factors became more difficult to overcome among parents in Colorado than in Washington. The reason for a differential effect by DU characterization is also unclear, although the intervention may do better at keeping children who start the recommended immunization schedule stay on that schedule (hence, higher proportion UTD on time) than it does at getting children caught up if they do not start on the schedule (hence, no change in mean percentage of DU).

### Limitations

There are limitations to this study. We found the intraclass correlation coefficient within the clinic was higher than expected (0.09 instead of 0.02). Because a higher intraclass correlation coefficient signifies greater similarity in outcomes within clinics, a larger sample size would have been needed (1550 parent participants with negative vaccine attitudes across 62 clinics) to have adequate power to accurately capture the variability between clusters and detect the desired effect size. Similarly, we observed higher SDs for our primary outcome than anticipated, which also reduced our power to detect the expected effect size. The demographics of our study sample, although reflective of Washington and Colorado, may not be generalizable to other US geographic areas. Additionally, our original intent to assess the fidelity of the intervention by videotaping a proportion of intervention visits was hindered by the COVID-19 pandemic and inability to have study staff in-person at clinic visits.

## Conclusions

In this cRCT of a tiered vaccine communication strategy, we observed no effect on vaccine uptake among children of parents with negative vaccine attitudes. The effect of the intervention, however, appeared to vary by state. Additional work is needed to understand the specific contributions of each component of the PIVOT with MI intervention on childhood immunization status and what other clinician communication strategies may be effective at improving vaccine uptake of children of parents with negative vaccine attitudes.

## Notes

Supplement 1.
Trial Protocol

Supplement 2. **eTable 1.** The Short Form of the Parent Attitudes about Childhood Vaccines (PACV) Survey **eTable 2.** Vaccine Codes Used for Analysis **eTable 3.** Specific Parameters for Determining Days Under-Immunized at 19 Months, 0 Days **eTable 4.** Results of Covariate-Constrained Randomization **eAppendix.** Training Curriculum for Clinicians (MD/DO, ARNP, PA-C) at Intervention Clinics
eReferences

Supplement 3.
Data Sharing Statement
