# Supplementary material for: Tiered Clinician Vaccine Communication Strategy to Improve Childhood Vaccine Uptake: A Cluster Randomized Clinical Trial
Source: JAMA Netw Open. 2025 Apr 30;8(4):e257814. doi: 10.1001/jamanetworkopen.2025.7814 (PMC12044505; doi:10.1001/jamanetworkopen.2025.7814)
Supplement: Supplement 1. — Trial Protocol [file jamanetwopen-e257814-s001.pdf]

Presumptively Initiating Vaccines and Optimizing Talk with Motivational Interviewing (PIVOT with MI)

COMIRB Protocol #17-1274
PI: O'Leary
V8. 5/6/2021

SPECIFIC AIMS

Though overall vaccination coverage of children in the US remains high,[1,2] sustaining current coverage levels is increasingly tenuous. **Public trust in vaccines has eroded,[3] nonmedical exemptions from kindergarten-entry vaccine requirements have increased,[4,5] and the proportion of parents refusing or delaying childhood vaccines has grown significantly.[6,7]** This has resulted in a persistent and prominent threat to individual and public health from large clusters of unvaccinated individuals that facilitate the transmission of vaccine-preventable disease.[8,9] Interventions to mitigate this threat are needed.[10]

Pediatric primary care providers are critical to efforts to stem the rise in childhood vaccine refusal and delay.[11] Parents cite pediatric providers as the most important influence on their vaccine decision-making[12] and provider communication behaviors used with parents during vaccine conversations at health supervision visits can influence parental vaccine acceptance.[13-15] Opel et al. have shown in observational work that when providers initiate their recommendations of childhood vaccines using a presumptive format (e.g. *"Well we have to do some shots."*) rather than a participatory one (e.g. *"How do you feel about vaccines today?"*), fewer parents—including vaccine-hesitant parents (VHPs)—voice resistance to the vaccine recommendation[14] and have increased odds of accepting all the recommended vaccines by visit's end.[16]

Independently complementing Opel et al.'s work, O'Leary et al. have identified Motivational Interviewing (MI) as an effective strategy for increasing compliance with provider vaccine recommendations. In a large, cluster randomized controlled trial (RCT) that focused on adolescent vaccinations, providers were educated on how to use MI with parents who verbally resisted vaccine recommendations. This intervention increased vaccine uptake and improved physician self-efficacy in vaccine discussions with VHPs.

While both a presumptive communication format for initiating vaccine recommendations and MI each hold promise as individual strategies for increasing childhood vaccination, combining the two using a tiered approach could have an additive effect on vaccine acceptance. We propose a novel and innovative provider vaccine communication strategy —**Presumptively Initiating Vaccines and Optimizing Talk with Motivational Interviewing (PIVOT with MI)**— in which providers use a presumptive format to initiate the childhood vaccine recommendation with all parents followed by use of MI with parents who voice resistance to that recommendation. **The main objective of the proposed study is to evaluate the impact of the PIVOT with MI intervention on child immunization status.**

The specific aims and hypotheses of this proposal are:

Aim 1: To develop the PIVOT with MI intervention provider training program. We will use an iterative process involving provider feedback through sequential focus groups to develop a multifaceted and feasible provider training program of the PIVOT with MI strategy with scalable components for dissemination.

Aim 2: To evaluate the impact of the PIVOT with MI intervention on child immunization status at 19 months of age. We will conduct a pragmatic cluster RCT using covariate-constrained randomization to balance key covariates in 24 practices from 2 practice-based research networks in Washington and Colorado. Providers in intervention practices will receive the PIVOT with MI intervention training program and providers in control practices will give usual care. All parents of newborns at intervention and control practices will be administered a short-form version of the validated Parent Attitudes about Childhood Vaccines survey[17,18] over an 18 month enrollment period to identify VHPs. Our primary outcome will be the mean percentage of days under-immunized for the 22 doses of 8 vaccines recommended from birth to 19 months among children of VHPs. Immunization data will be obtained from the Washington and Colorado immunization information systems. We hypothesize that children of VHPs at intervention practices will be less under-immunized than children of VHPs at control practices.

Aim 2a: To assess the fidelity of the PIVOT with MI intervention. We will videotape a proportion of provider visits with VHPs at intervention practices to observe adherence to the intervention. We hypothesize that intervention providers will use PIVOT with MI techniques in >80% of encounters.

<u>Aim 3</u>: To evaluate the impact of the PIVOT with MI intervention on parent and provider experience. We will use a cross-sectional survey design to assess visit experience of VHPs at intervention and control practices. We will conduct pre-, interim - and post-intervention surveys of intervention and control providers to assess time spent and self-efficacy in discussing vaccines with VHPs. We hypothesize that VHPs at intervention visits will have a higher-rated experience than VHPs at control practices and intervention providers will spend less time and have improved self-efficacy in vaccine discussions with VHPs compared to control providers. Additionally, due to implications of COVID-19 on research and health care delivery, a new survey will better help the study team understand the extent of the impact of COVID-19 on the delivery of health supervision visits at our pediatric clinics, specifically on infant vaccine delivery and uptake.

The proposed study uses a rigorous design to test an innovative, evidence-based provider communication strategy to address a critical public health problem. We plan to use our results to inform future dissemination and implementation work conducted within the American Academy of Pediatrics' Pediatric Research in Office Settings network and the American Academy of Family Physicians' National Research Network.

RESEARCH STRATEGY

A. SIGNIFICANCE

The reduction of vaccine-preventable diseases (VPDs) is a top public health priority in the US today.[19] Parental refusal or delay of childhood vaccines is a key contributor to the persistence of outbreaks of VPD[20] and is associated with higher inpatient admission and ED utilization rates,[21] increased morbidity,[22-24] and death.[25] There is therefore renewed emphasis on sustaining and improving childhood vaccine coverage.[11,26]

Vaccine-hesitant parents (VHPs) are an important population for targeted interventions aimed at improving vaccine acceptance.[27] VHPs are a large (10-30%),[13,17,18,28,29] heterogeneous group of parents who refuse and/or delay at least one recommended vaccine for their child.[30] Compared to the small minority (1-2%) of parents who completely reject vaccines,[31] VHPs hold less negative attitudes and beliefs towards vaccines[13,30,32-34] and therefore are more likely amenable to behavior change.[27]

Provider communication with VHPs is critical to achieving this behavior change.[35] VHPs consider their child's pediatric provider to be a key information source in their decision-making about vaccines and their child's health,[13,15,30,32,36] with a proportion of initially hesitant parents reporting that they changed their mind about delaying or refusing a vaccine after their child's provider addressed their concerns, provided them with additional information, or gave them reassurance.[13,15] **Consequently, how providers discuss and recommend vaccines is significant.** The quality and presence of a provider's recommendation has been associated with increased uptake of childhood and adolescent vaccines.[37-47] <u>Moreover, Opel et al. have found in observational work that the communication format used to initiate the vaccine recommendation is influential: a presumptive (e.g. *"Well we have to do some shots."*) rather than a participatory (e.g. *"How do you feel about vaccines today?"*) format was associated with increased parental vaccine acceptance of childhood vaccines.</u>[14,16] In a recent randomized controlled trial (RCT) based on this work, there was a 5.4% (95% confidence interval [CI]: 1.1%–9.7%) increase in HPV vaccine uptake in adolescents attending clinics whose providers received training on the presumptive format for initiating the HPV vaccine recommendation compared to control clinics whose providers received no training, with no difference in uptake between control clinics and clinics whose providers received training on use of the participatory format.[48] This evidence strongly suggests that provider use of the presumptive format can increase parental acceptance of vaccines.

Several barriers exist to integrating these communication strategies into health supervision visits. First, a presumptive format for initiating the vaccine recommendation has been associated with decreased odds of parents rating their visit experience highly,[16] raising concerns that any short term gain in parental vaccine acceptance with use of this strategy may be negated by decreased vaccine uptake over the long term.[49] Second, a substantial percentage of providers lack confidence in communicating with parents who have concerns about vaccines[28] or perceive their discussion will do little to change a parent's mind.[50] **To address these barriers, O'Leary et al. have explored the feasibility and efficacy of using Motivational Interviewing (MI) when parents resist the vaccine recommendation.** MI is a well-established, evidence-based, patient-centered framework for behavior change[51-68] that is effective even when delivered in a single session.[54,62] MI's 3 essential elements—having a conversation, leveraging inherent motivation for behaviors, and making the conversation person-centered—make it well adapted for use with VHPs given their known communication preferences regarding vaccines.[15,30] <u>In a large RCT, O'Leary et al. found that provider use of MI in discussions with parents who verbally resisted the HPV vaccine recommendation resulted in increased HPV vaccine acceptance and improved provider perceptions of their ability to influence parental vaccine decision-making.</u>

A major gap in our understanding of how to effectively communicate with parents in the early childhood vaccine context is the lack of experimental evidence for specific strategies. Though experimental evidence has emerged in the adolescent vaccine context, data in the early childhood vaccine context supporting specific communication strategies like the format of the vaccine recommendation remains observational and from small sample sizes.[10] There has only been one randomized trial of a provider communication strategy designed to improve parental hesitancy in the childhood vaccine context, but it did not test the impact of the strength, format, or presence of a provider's vaccine recommendation.[28]

We propose to conduct a cluster RCT among 24 practices in 2 practice-based research networks (PBRNs) in Washington and Colorado to evaluate the effect of a provider communication strategy that combines the 2 communication strategies studied by Opel et al. and O'Leary et al.—**the Presumptively Initiating Vaccines and Optimizing Talk with Motivational Interviewing (PIVOT with MI) intervention**—on child immunization status as well as on parent and provider experience. The PIVOT with MI intervention involves providers using the presumptive format to initiate the childhood vaccine recommendation with all parents followed by use of MI if a parent verbally resists the recommendation. This tiered approach involving 2 complementary communication strategies has the potential to have the additive effect of improving parental vaccine acceptance and provider self-efficacy while preserving a highly-rated parent visit experience. This proposal is therefore responsive to existing gaps and barriers to communicating with parents about vaccines by not only addressing the need for experimental evidence demonstrating the effectiveness of specific communication strategies in the childhood vaccine context,[10] but also addressing concerns about parent visit experience with use of the presumptive format alone and provider confidence in communicating with VHPs.

B. INNOVATION

This study is innovative in 3 ways. **First, we are seeking to shift current practice by testing the effectiveness of a novel provider-based communication intervention in improving childhood vaccine uptake.** At present, the lack of experimental evidence for specific vaccine communication strategies in the childhood vaccine context has hindered the development of a standardized approach to communicating with VHPs[69,70] and hampered progress in efforts to address vaccine hesitancy.[71]

Second, the PIVOT with MI intervention applies innovative insights from behavioral economics and decision psychology, two fields of emerging importance in understanding human behavior,[72] to the vaccine encounter.[73] In particular, the presumptive initiation format leverages choice architecture as a means to achieving a desired goal: whether a decision is presented as an opt-in or opt-out impacts the choice made, with the large majority of choosers sticking with the default decision made for them.[74,75] The primary driver behind this behavior is status quo bias, a cognitive bias inherent to human decision-making that results in an aversion to actively changing a decision that has already been made.[76]

Third, we are the first to propose and test the use of MI techniques when parents resist the presumptive (i.e. opt-out) recommendation. Interpersonal communication dynamics have been shown to be a key influential factor affecting health behavior,[77] but most resources developed for providers when they encounter VHPs do not account for these dynamics. Instead, these resources are primarily based on the Information Deficit Model,[78] which assumes that misperceptions are due to a lack of knowledge and that the solution is to provide more information. Yet, there is robust literature across disciplines to suggest that simply providing information often does not lead to people changing their views and may even create a dynamic in which a parent is actually less receptive to information a provider may impart.[79,80] By training providers to use communication techniques based on the principles of MI when parents voice resistance to the presumptively formatted vaccine recommendation, we seek to avoid this negative dynamic and thus better equip providers to change the vaccination behavior of VHPs.

C. APPROACH

C.1. Preliminary Studies

The following list of studies is intended to provide evidence of the rationale for the PIVOT with MI intervention and the study team's expertise in the methods proposed in the current application. Please also note that the study team has expertise in organizing and conducting provider focus groups to obtain feedback on vaccine materials and intervention design, as proposed in Aim 1.[81-83]

C.1.a. Development and Evaluation of the Parent Attitudes about Childhood Vaccines Short Form (PACV-SF) Survey (Opel, PI; Taylor, Co-I; Zhou, Co-I): The PACV is a 15-item survey designed to identify VHPs that takes <5 minutes to complete and reads at the 6 – 7th grade level. We developed the PACV using a standard iterative process.[84] The PACV's validity was then evaluated in 2 separate studies and found to have both construct and predictive validity.[17,18] Ongoing evaluations of the PACV support its validity in

different geographic populations[29,85,86] and clinical settings.[87] In collaboration with investigators at Texas Children's, we have also developed a Spanish version of the PACV using the 4-step WHO guidelines for translation of instruments.[88]

**Table 1.** PACV-SF: Positive if ≥2 hesitant responses		
No.	Item	Hesitant Response
1	Have you ever delayed having your child get a shot for reasons other than illness or allergy?*	Yes
2	How concerned are you that a shot might not prevent the disease?	Very concerned, somewhat concerned, or not sure
3	Overall, how hesitant about childhood shots would you consider yourself to be?	Very hesitant, somewhat hesitant, or not sure
4	I trust the information I receive about shots.	Strongly disagree, disagree, or not sure
*Only delay of the birth dose of the hepatitis B vaccine would be relevant for parents of children ≤2 months old, as was used and evaluated in a prior study[18]		

Opel and Zhou also conducted a secondary analysis of predictive validity data to identify a short form of the PACV (PACV-SF; Table 1). Different candidate item combinations and score thresholds were optimized on 4 criteria: 1) reflection of the major sub-domains of vaccine hesitancy; 2) specificity [to minimize false positives]; 3) sensitivity [to maximize true positives]; and 4) minimize the total number of items on the PACV-SF. Construct validity data was then used as a cross-validation sample to assess PACV-SF performance.

We found that hesitant responses on ≥3 of 4 items optimized our 4 criteria: it had an 85% (95% CI: 75, 92) sensitivity and 96% (95% CI: 94, 98) specificity in the original sample and 90% (95% CI: 79, 96) sensitivity and 95% (95% CI: 90, 98) specificity in the validation sample for identifying respondents with PACV scores ≥50. *We concluded that the PACV-SF is valid for identifying VHPs and will be used to do so in the proposed study.*

C.1.b. Impact of a Provider Communication Strategy on Parental Vaccine Hesitancy Towards Childhood Vaccines (Opel, Co-I): Opel et al. conducted a cluster RCT of 56 pediatric and family medicine clinics in the greater Seattle metropolitan area to test the effect of a provider communication strategy on parental vaccine hesitancy.[28] There were 26 clinics in the control arm in which parents of newborns received usual care and 30 clinics in the intervention arm in which providers received training on a communication strategy called: "Ask, Acknowledge, and Advise." The centerpiece of this strategy was an algorithm in which providers were first encouraged to begin the vaccine recommendation by asking parents what questions they had about the immunization schedule, then acknowledging parental vaccine concerns, and finally, advising parents in a manner specific to their concerns. The intervention training included a well-attended (68% of providers) didactic session at baseline that incorporated videos of providers modeling the communication strategy. Providers in intervention clinics also received paper materials detailing the framework as well as monthly e-mail newsletters and a link to the study website that included a webinar version of the training.

Our primary outcome was the change in the proportion of parents who were VHPs (as measured by the PACV) at recruitment after the birth of their child and follow-up when the child was 6 months old. We found that among all enrolled parents, 11.3% were VHPs. The proportion of VHPs at baseline and follow-up changed from 9.8% to 7.5% in intervention clinics and 12.6% to 8.0% in control clinics (adjusted odds ratio [aOR]: 1.22; 95% confidence interval [CI]: 0.47-2.68). *We concluded that this provider communication strategy was not effective in reducing parental vaccine hesitancy. This trial, in part, informed the work described below in C.1.c. where we found that the opposite approach to the 'ask' strategy—a presumptive initiation of the vaccine recommendation—was more effective. It also provides evidence of our expertise in recruiting for and conducting a pragmatic cluster RCT of a provider vaccine communication intervention.*

C.1.c. Identification of Initiation Format as a Relevant Provider Vaccine Communication Practice (Opel, PI; Taylor, Co-I; Robinson, Co-I; Zhou, Co-I; Hofstetter, Co-I): In a pilot cross-sectional observational study to characterize the provider-parent vaccine discussion, Opel et al. videotaped vaccine discussions (N=24) between VHPs of children 2 – 15 months old and 9 different pediatric providers from 6 practices in the Seattle, WA area belonging to the Puget Sound Pediatric Research Network (PSPRN).[89] We identified 6 provider communication practices that appeared relevant to parental vaccine decision-making, including how providers initiated the vaccine discussion. In our sample, we found providers used 2 ways to initiate the vaccine discussion: a presumptive format, in which they presupposed that shots would be given (e.g. *"Well we have to do some shots."*), or a participatory format, in which providers encouraged parental input and agency in the vaccine decision (e.g. *"What do you want to do about shots?"*).

In a follow-up cross-sectional study to quantify how the 2 initiation formats were associated with parental vaccine acceptance and visit experience, Opel et al. enrolled 16 pediatric providers from primary care practices in PSPRN and other Seattle area practices (N=9) and videotaped 111 of their vaccine discussions with parents of 1 – 19 month old children.[14,16] We enrolled both hesitant and non-hesitant parents using the validated PACV survey.[17,18,84] We found that parents had significantly higher odds of verbally resisting vaccine

recommendations immediately after the provider initiated the vaccine discussion if the provider used a participatory rather than a presumptive approach (aOR: 17.5; 95% CI: 1.2 – 235.5). Furthermore, a participatory (vs. presumptive) approach was associated with decreased odds of accepting all vaccines by visit's end (aOR: .04, 95% CI: .01, .15) but increased odds of a highly rated visit experience (aOR: 17.3; 95% CI: 1.5, 200.3). In a secondary analysis of visits that contained a discussion of influenza vaccine, we also found parents had increased odds of accepting influenza vaccine at visit's end when providers initiated their influenza vaccine recommendation using a presumptive (vs. participatory) format (aOR 48.2, 95% CI: 3.5-670.5).

In a second follow-up study, we assessed whether a participatory format—though associated with decreased vaccine acceptance *after a single visit* compared to a presumptive format—would be associated with increased parental vaccine acceptance *over time*. We conducted a longitudinal prospective cohort study in which we enrolled VHPs (defined as PACV score ≥50) of newborns (N=72) prior to their child's 2 month health supervision visit at Group Health Cooperative (GH), an integrated health care system based in Seattle, WA. After their child's 2, 4, and 6 month visits, we asked parents what format their child's provider used to begin the vaccine discussion (categorized as presumptive, participatory, or neither) utilizing a validated instrument for parent self-report of provider initiation format. We used GH records and Washington State Immunization Information System (WAIIS) records to assess child immunization status at 8 months of age and after each visit. We found that exclusive (vs. non-exclusive) use of a participatory format was associated with a child being 37.2% (95% CI: 9.8, 64.6; *P*=.009) more days under-immunized at 8 months of age and visit-specific use of a participatory (vs. presumptive) format was associated with a child being 14% (95% CI: 1.4, 26.6; *P*=.03) more days under-immunized after each visit. **Through these series of projects, we concluded that the presumptive (vs. participatory) communication format for initiating childhood vaccine discussions was positively associated with parental vaccine acceptance and negatively associated with parent visit experience, and that use of a participatory (vs. presumptive) format did not improve vaccine uptake over time.** *These studies provide a basis for the PIVOT with MI intervention and demonstrate our expertise in a number of methodological areas relevant to the current proposal including qualitative and quantitative research, measurement of our primary study outcome of days under-immunized, use of immunization registry data, and conducting research within a PBRN. These studies, however, are limited by their observational design, small sample sizes, and inability to make conclusions regarding the association of initiation format on immunization status beyond 8 months of age. The current proposal addresses these limitations by using an RCT design to test the PIVOT with MI intervention on immunization status at 19 months of age.*

C.1.d. The Physician Communication (PCOM) Study (Dempsey, PI; O'Leary, Co-I): O'Leary et al. conducted a cluster RCT involving 16 Colorado Children's Outcomes Network (COCONet) practices and 125 providers to assess the effect of the PCOM intervention on HPV vaccine uptake and on provider self-efficacy in communicating with parents about HPV vaccination. The intervention involved 4 intervention components: HPV fact sheets, the iVac website, and a decision aid for parents as well as in-person and video training on MI for providers. This provider training focused on MI processes made relevant to the HPV vaccination context, including engagement (seeing the vaccination decision through the parents eyes), collaboration (avoiding parent confrontation by inviting parents to examine their position from other points of view), and evocation (eliciting parents inherent motivation by evoking and reinforcing change talk [vaccine acceptance]).
Using immunization data obtained from practices' electronic health records (EHRs) and the Colorado Immunization Information System (CIIS), we found intervention practices had 8 percentage point higher increases from baseline in HPV vaccine series initiation than control practices (Table 2). **To our knowledge, this is the first evidence for the effectiveness of MI techniques to change parental willingness to vaccinate.** Provider surveys and qualitative interviews revealed that, of the 4 intervention components, MI was the most frequently used and most useful, with 72% of providers reporting using it frequently 9 months after the training occurred, and 79% of providers reporting it to be useful in facilitating HPV vaccine conversations with hesitant parents. Importantly, providers using MI reported spending *less* time discussing the vaccine during visits with VHPs compared to prior to the training (P<0.001) and had improved self-efficacy for changing HPV vaccine hesitant parents' minds about vaccination (P=0.009). *We concluded that MI is an effective communication strategy with parents who resist a provider's HPV vaccine recommendation. These results provide a basis for the PIVOT with MI intervention and evidence of our expertise in the methodological areas relevant to the current proposal, including pragmatic*

Table 2. PCOM Trial Results: Percentage point (PP) increases from baseline (BL) in HPV vaccine initiation

HPV Vaccine Series Initiation (age)	**Intervention**			**Control**			**P**
	BL	Post	Δ PP	BL	Post	Δ PP	
11-12 years old	38%	51%	**+13%**	41%	46%	**+5%**	0.003
13-17 years old	23%	32%	**+9%**	30%	27%	**-3%**	<0.001

trial design and implementation within a PBRN, assessment of practice, patient and provider factors associated with intervention deployment, and analysis of EHR and immunization registry data.

C.2. Associated Research Partners

The setting for this proposal is 2 independent and well-established regional pediatric PBRNs in 2 states (Washington and Colorado). Each of the practices in these PBRNs participate in CIIS and WAIIS (the immunization registries for each state), the main source of vaccine data for the project.

C.2.a. Puget Sound Pediatric Research Network (PSPRN): PSPRN is a regional PBRN founded in 1996 and currently directed by Taylor. PSPRN includes 6 core practices and 12 supplementary practices that participate in studies on an ad-hoc basis. PSPRN practices include >100 pediatricians, are situated in varied areas (urban, suburban, and rural), and encompass different practice settings (university-based, community-hospital based, private, and multispecialty group). Opel has conducted preliminary studies for this proposal with PSPRN (see *C.1.c.*) and Taylor has conducted large studies with PSPRN including RCTs involving 500 child participants and observational studies with over 1,000 patients.

C.2.b. Colorado Children's Outcomes Network (COCONet): COCONet is Colorado's pediatric PBRN and is led by O'Leary. O'Leary and Dempsey have conducted preliminary study C.1.d. within COCONet. Through this and other studies, O'Leary has developed excellent working relationships with COCONet practices and well-established mechanisms to access immunization data from these practices' EHRs. COCONet currently has 60 member practices with >300 providers and several hundred thousand pediatric patients. There is a broad spectrum of both public and private practices within COCONet, with many of the practices having high proportions of VHPs as well as socio-economic and racial/ethnic diversity. O'Leary and other investigators within ACCORDS have been working with practices within the network on research projects for >15 years.

Figure 1. Research Studies Flow Diagram

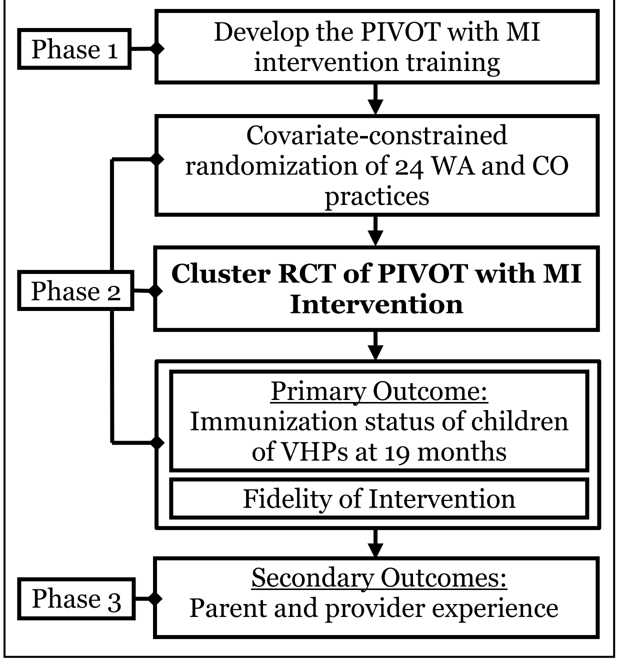

C.3. Overview of Study Activities

This study aims to assess the impact of the PIVOT with MI intervention on child immunization status and can conceptually be divided into 3 phases (Figure 1). Phase 1 involves an iterative process to develop, review, and revise the PIVOT with MI provider training components, including the use of provider focus groups for input on the usability, understandability, and acceptability of training materials. Phase 2 is a cluster RCT using covariate-constrained randomization to balance key covariates in 24 PSPRN and COCONet practices (excluding COCONet practices that participated in the PCOM study; see *C.1.d.)* to assess the effect of the PIVOT with MI intervention on the immunization status of children of VHPs at 19 months of age. Phase 3 involves the assessment of secondary outcomes, including parent visit experience and provider self-efficacy.

Figure 2. Difficulty by Motivation Matrix

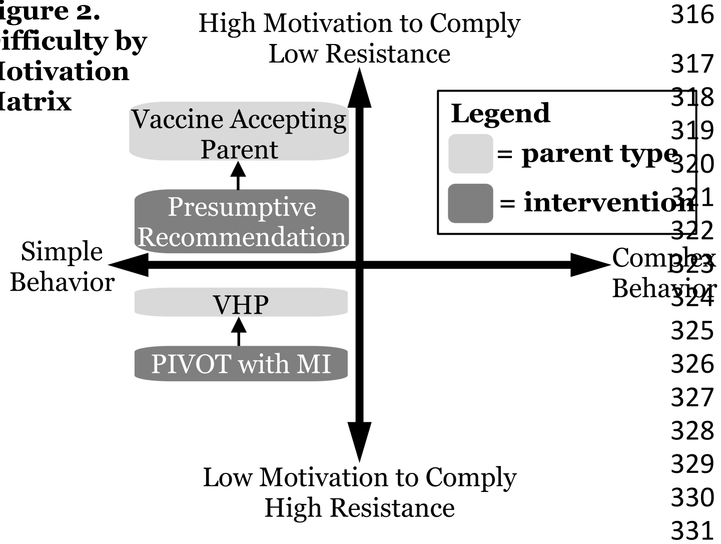

C.4. Conceptual Framework

In addition to its theoretical foundations in behavioral economics and decision science (see *B.*), the PIVOT with MI intervention is grounded in a conceptual framework by Resnicow et al. called the "Difficulty by Motivation" matrix (Figure 2).[90] This matrix, which can be applied to both health behaviors and health behavior interventions, parses behaviors or interventions into 4 quadrants. Quadrants represent properties of the health behavior or intervention being considered (x-axis) and individual level factors including an individual's motivation and/or competence to comply with the recommended behavior or intervention (y-axis). Vaccination can

be categorized as a simple health behavior given it is an intervention with a high certainty of low risk and high benefit. Vaccine accepting parents reside in the upper left quadrant given their high motivation to comply with vaccination. Interventions to promote compliance with vaccination simply need to match this motivation, such as using a presumptive format for initiating the vaccine recommendation that leverages vaccination as a normative behavior. In contrast, VHPs reside in the lower left quadrant with high resistance and low motivation to comply. As such, interventions to promote vaccination with VHPs need to be more robust. Following a presumptive initiation of the vaccine recommendation, VHPs are more likely to verbally resist vaccination signaling to the provider that the parent is a VHP and is in need of a more intense intervention. This recognition can serve as a **"pivot point"** that triggers the provider to shift from the normative action of a presumptive recommendation to MI in order to try and leverage a parent's intrinsic motivations for doing what they perceive is best for their child.

C.5. Aim 1: To develop the PIVOT with MI intervention provider training program.

C.5.a. Overview: The observational studies of Opel et al. (see *C.1.c.*) and the intervention study of O'Leary et al. (see *C.1.d.*) identify complementary strategies that can be used in a tiered manner to increase vaccine uptake without compromising parent visit experience. The combination of these 2 communication strategies is a novel and innovative, yet evidence-based, approach to addressing parental refusal or delay of vaccines in the pediatric outpatient setting. We will use an iterative process to develop, review, and revise the content, format, structure, and processes involved in implementing the PIVOT with MI intervention. We will also conduct focus groups of providers at several points during this development to further enhance the usability, understandability, and acceptability of the training materials.

C.5.b. Design: The PIVOT with MI intervention that we propose to develop will be multifaceted[91-93] and include several approaches based on adult learning theory[94] previously found to be effective in changing provider behavior.[95,96] These approaches include interactive and tailored educational outreach,[97] provider rehearsal and coaching,[98] audit and feedback,[99] booster learning sessions,[98] and change agents.[100] Specifically, we propose that the PIVOT with MI intervention include the following components: **1) an online video module** introducing the PIVOT with MI communication strategy, its rationale, and a model vaccine visit utilizing it; **2) an in-person interactive provider training** session that will include a) a brief didactic session on vaccine hesitancy, how the PIVOT with MI strategy addresses vaccine hesitancy, and practice data on vaccination coverage and vaccine hesitancy prevalence, b) baseline assessments of provider skills using the presumptive format and MI, and c) modeling of the PIVOT with MI intervention followed by provider rehearsal through role-playing and coaching by the study team; **3) provider reference sheets** that provide brief and accessible summaries of the communication behaviors the comprise PIVOT with MI, along with example statements for key steps in the PIVOT with MI communication strategy; **4) two refresher trainings** at 6 and 12 months after the start of the intervention that will include a question and answer (Q+A) session regarding barriers to implementing the PIVOT with MI intervention followed by role-playing and coaching. The 12 month refresher training will also include a review of select videotaped encounters of intervention providers with VHPs to provide feedback for how to improve incorporation of PIVOT with MI into the vaccine discussion; **5) study champions** will be identified among staff at each intervention practice to serve in a role of advocacy, information and implementation support for the study throughout the intervention period. The study champion will routinely solicit feedback from intervention providers regarding the PIVOT with MI intervention and liaise with the study team at regular intervals to communicate and help address implementation issues.

C.5.c. Provider Focus Groups and Semi-Structured Interviews: To inform intervention development, we will conduct one 90-minute provider focus groups involving 5-10 providers (1 each in Washington and Colorado) at the start of the development process to elicit provider input on the format, length, and content of the proposed intervention components as well as to identify design and acceptability issues. We will subsequently conduct a) individual semi-structured interviews with a unique set of 5-10 providers at approximately month 6 of the 12-month training material development period and b) second focus group at approximately month 9 that includes another unique set of 5-10 providers. These interviews and second focus group will each to allow for review, input, and sequential refining of draft training content and materials. Vetted materials will be finalized and ready for implementation by the start of year 2 of the project.

Recruitment of providers for interviews and focus groups will occur among primary care practices that will not be enrolled in the study to avoid contamination. Specifically, providers from 3 practices in Colorado that were in the intervention arm of the PCOM trial will be targeted, as these providers will be familiar with the use of MI for vaccine conversations and will be valuable resources for suggestions on adapting these techniques for use with parents of newborns. Providers will be provided with a $100 gift card for participation.

Focus groups and interviews will be conducted by a trained moderator using a guide developed by the study team (Appendix 1). These focus groups and interviews will be in-depth and semi-structured, utilizing a

combination of broad, open-ended questions and more specific probes. Focus groups will occur in person and individual interviews will occur over the phone with materials needed for review disseminated ahead of time. A co-moderator may take detailed notes during each in-person session or phone interview. Each focus group will be audiotaped and/or transcribed.

C.5.d. Iterative Development Process: The study team will meet via conference call every 2 weeks for the first 12 months of the proposed project to develop the intervention. Study team members will first review moderator notes and transcribed audiotapes from the initial provider focus groups to inform the initial drafts of the components of the intervention. Opel, Taylor, Robinson, and Hofstetter will develop the training content for the presumptive initiation format that will include the incorporation of communication examples collected as part of preliminary work (see *C.1.c.*). O'Leary, Dempsey, and Garrett will develop the MI content by adapting MI training materials from the PCOM intervention (see *C.1.d.*). A draft video module will be developed in collaboration with CU Productions (*see Facilities and Other Resources*), a video production team used by study investigators for PCOM (see *C.1.d.*). The entire study team will review all draft content and revise iteratively to fit desired formats, time allotments, and study objectives. After presentation of the draft materials to providers during the 2nd and 3rd focus groups, the study team will again review moderator notes and transcribed audiotapes to iteratively identify and address design, content, and format issues to intervention materials.

C.5.e. Online Video Module Post-Production: In the final year of the study period, the study team will again collaborate with CU Productions to adapt the video module used in the PIVOT with MI intervention in order to support its implementation in more general settings. This will involve incorporating lessons learned during the course of the study, creating a new introduction to the module that it is applicable to non-research settings, and developing follow-up video modules that simulate the in-person refresher trainings.

C.5.f. Strengths and Limitations: The PIVOT with MI intervention will be multifaceted and intensive to maximize provider behavior change, will include scalable elements (online video module and provider references sheets) to promote implementation, and will be developed by a study investigator team comprised of general pediatricians, health services researchers, and communication experts with assistance from an experienced video production team. More than 6 focus groups may be needed to refine training content and materials. If so, additional focus groups will be conducted.

Figure 3. Study Design

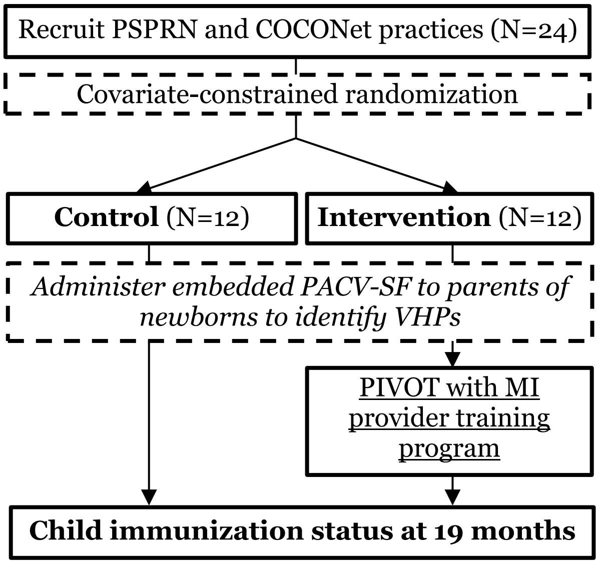

C.6. Aim 2: To evaluate the impact of the PIVOT with MI intervention on child immunization status at 19 months of age.

C.6.a. Overview and Design: We propose to conduct a cluster RCT (Figure 3). We will randomize PSPRN and COCONet practices (N=24) to control and intervention arms using a covariate-constrained randomization approach. All parents of newborn infants receiving health supervision at a study practice will be screened for vaccine hesitancy at their child's initial visit using the PACV-SF (see *C.1.a.)* in order to identify VHPs, the primary study population. Providers at intervention practices will receive the proposed 5-component PIVOT with MI provider training program (see *C.5.b.*). Providers at control clinics will give usual care. A subset of VHPs at intervention clinics who give us permission to contact them will have their child's health supervision visit videotaped to assess intervention fidelity. Our main outcome will be child immunization status at 19 months expressed as the mean percentage of days under-immunized from birth to 19 months.

C.6.b. Setting and Population: Two regional PBRNs (PSPRN and COCONet) from 2 states (Washington and Colorado) will serve as the study setting (see *C.2.*). Both Washington and Colorado are ideal settings to conduct a study of an intervention designed to improve immunization rates among VHPs because both have a high prevalence of VHPs, with each ranking in the highest quintile among US states with respect to the proportion of parents claiming non-medical exemptions for their child from required school-entry vaccines.[2]

C.6.c. Eligibility: Providers will be eligible if they belong to PSPRN or COCONet. All English- and Spanish-speaking parents with a newborn infant <1 month old receiving health supervision at a participating practice during the intervention period will be eligible. VHPs, defined as those who have a positive PACV-SF *(see C.1.a.),* will constitute our primary study population because we are most interested in changing their vaccination behavior.[10,27] VHPs at intervention practices who indicate on the PACV-SF that study staff can contact them about participation in additional study activities will be eligible for the fidelity study (Aim 2a).

C.6.d. Practice Recruitment: We have commitment from the PSPRN director and COCONet steering committee to use each network to recruit practices (*see letters of support from Taylor and COCONet Steering Committee*). Upon notice of award, Opel and Taylor will contact all PSPRN practices (N=18) and O'Leary and Dempsey will contact COCONet practices that didn't participate in the PCOM study (N=50; see *C.1.d.*) to invite them to participate. Among interested clinics, meetings will be arranged between the study team and practice representatives, including any relevant decision makers such as office managers and managing partners. At these meetings, the timeline of the study will be reviewed and questions will be answered. Both PRBNs are large enough to accommodate declination from some practices due to unforeseen circumstances, such as provider turnover or new EHR implementation, and still meet sample size goals. Should the need arise to recruit more practices, Seattle- and Denver-area practices that are not part of PSPRN or COCONet will be approached. Both PIs have successfully included non-PBRN practices in past studies (see *C.1.b.-d.*).

We will consider a practice enrolled when clinic leadership agrees to participate. After a practice is enrolled, Business Associates Agreements (BAA) and Data Use Agreements (DUA) will be drafted and signed as appropriate. Leaders at enrolled clinics will be asked to encourage provider participation and attendance at study kick off meetings (all practices) and communication trainings (intervention practices only). As an incentive for providers to participate in the study and for intervention providers to be engaged through the project period, we will work with the American Board of Pediatrics and the American Board of Medical Specialties to get this project certified for Maintenance of Certification (MOC) Part 4 credit. All board-certified physicians are required to meet certain requirements to maintain their certification,[101] and prior work has shown that getting projects certified for MOC credit increases engagement among providers.[102,103] Study certification with MOC credit will also facilitate post-award dissemination of the proposed intervention and receipt of MOC credit by providers at control practices, since the MOC certification can be modified so that any eligible US physician could receive credit by completing the educational module and meeting other requirements. The study team has been successful in getting past projects certified for MOC credit (see *C.1.d.*) and using MOC credit as an incentive to increase provider study participation.

C.6.e. Covariate-constrained Randomization: Practices will be the unit of randomization. A key issue in cluster-randomized trials is the possibility of covariate imbalance in practices assigned to different treatment arms.[104-108] Thus, practices will be randomly assigned to assure balanced comparison groups using optimized probability sampling to the intervention or control arm in the following manner. From the pool of practices that agree to participate in the study, pre-study information will be collected on several characteristics that may influence study outcomes: percent of VHPs, percent of Vaccines-for-Children [VFC]-eligible patients, number of pediatric providers, and practice immunization rates of children 19-35 months old. These pre-study variables will then be used to develop and evaluate a balance criterion, defined as the sum of the squared differences between standardized practice means on these variables.[105] All possible combinations of eligible practices in 2 arms (intervention and control) will be generated using a SAS macro program.[107] The distribution of the balance criterion for the 2 study arms will be used to define an acceptable set of study arms that are reasonably balanced in terms of the selected variables (minimum 10% of balance criterion).[105] From this set, one set will be chosen at random and used to randomly assign each practice to intervention or control arms. This process will be undertaken in both Colorado and Washington as separate randomization procedures. We have successfully used covariate-constrained randomization in C.1.d. and other studies.[109,110]

We will perform the following procedures to obtain pre-study information on these covariates:

C.6.e.1. Assessment of the proportion of VHPs at each practice: For a period of 2 months prior to the start of the PIVOT with MI intervention period, we will administer an English- or Spanish-language version of the PACV-SF (see *C.1.a.*) to all English- and Spanish-speaking parents with newborns upon check-in for their newborn's first visit at a participating practice. Practice staff will include the PACV-SF in the practice's standard intake paperwork that parents are asked to complete at this first visit. **We have utilized this design within COCONet practices in the past and have achieved nearly full participation even with a disclaimer on the survey stating its completion is voluntary and for research purposes.** This design will allow us to obtain a stable estimate of the proportion of VHPs in each practice as well as refine the procedures necessary at each clinic to successfully integrate the PACV-SF into practice workflow, as this screening process will continue throughout the intervention. Study staff will visit study practices at regular intervals to pick-up completed surveys to score them and conduct data entry. One staff member at each practice will receive a $100 gift card as a thank you for overseeing the implementation of PACV-SF screening.

C.6.e.2. Assessment of the proportion of VFC-eligible patients at each practice: The proportion of VFC-eligible patients at each practice will be determined in collaboration with the office manager at each

participating practice. In prior studies, office managers have been able to calculate this with a high degree of accuracy when compared to EHR or immunization registry data.

C.6.e.3. Assessment of the number of pediatric providers: We will determine the number of pediatric providers at each practice using the contact information provided at practice enrollment by practice leaders.

C.6.e.4. Assessment of the proportion of children who are up-to-date (UTD) on immunizations at each practice: We will conduct a retrospective analysis of UTD status for all recommended childhood vaccines among children 19-35 months old at each practice using vaccine administration data for each practice obtained from the two state's immunization registries (WAIIS and CIIS). The WAIIS and CIIS operate in accordance with nationally recommended standards for immunization registries, and all PSPRN and COCONet practices participate in CIIS or WAIIS. Both CIIS and WAIIS use birth certificate data to identify children and cover ≥95% of children <6 years old in their respective states.[111] Both CIIS and WAIIS[112] combine the immunization information from multiple sources into a consolidated, complete, and valid record of immunizations. Data quality assessments are done frequently. Offices are required to have <5% error rate in the registry, making these registries a highly accurate data source for assessing vaccine utilization.

C.6.f. Intervention: Intervention specifics will ultimately be determined through the iterative process detailed in Aim 1 (see *C.5.c.-d.*). Therefore, what follows is preliminary and will be modified. At baseline, providers at intervention practices will be electronically sent a link to the online video module to view at their convenience. Modules will be approximately 30 minutes and will be encoded with a viewing tracker that will enable study staff to monitor its completion. Providers who have not viewed the module within 2 weeks will be sent up to 3 reminders with additional links to the video module. The window for viewing the video module will be 30 days, and viewing the video will be required for provider to receive MOC Part 4 credit. Based on our previous study utilizing a baseline video module (see *C.1.d.*), we expect a ≥70% completion rate.

At the conclusion of the video module window, providers will be asked to attend a 90 minute interactive in-person training session led by study staff. Attendance will be required for MOC Part 4 credit. This baseline training session will involve a baseline assessment of provider skills with the communication techniques introduced in the module and teaching and reinforcement of the PIVOT with MI strategy using modeling, role-playing and coaching by the study team. Providers will also receive instructions on how to use the reference sheets developed in Aim 1. Each intervention practice will have its own in-person training session. For PSPRN practices, sessions will be led by Opel, Hofstetter, Robinson, and Garrett. For COCONet practices, sessions will be led by O'Leary, Dempsey, and Garrett. Based on our previous work, provider participation rate in this baseline training is expected to be ≥70% (see *C.1.b.* and *C.1.d.*). The intervention period will commence at the conclusion of this baseline training and last 36 months (constituting the 18 month parent recruitment period [see *C.6.k.*] and the 18 months after this period to allow for the last enrolled newborn to reach 19 months old).

Six months into the intervention period, providers will receive a 60 minute in-person refresher training led by the same study team members. This will take a similar format to the initial training but will start with providers sharing their experiences with the PIVOT with MI intervention over the prior 6 months, with demonstrations from each provider of a difficult vaccine conversation with feedback from the MI trainer. At 12 months into the intervention period, we will have a second 90 minute in-person refresher training that will also include viewing 3-5 selected videotaped encounters of 2, 4 and/or 6 month health supervision visits involving intervention providers (with their permission) from the preceding 12 months (see *C.6.g.*) to illustrate "dos and don'ts" of the PIVOT with MI strategy. We will ask providers at the refresher training what they thought went well and what could be improved after watching each videotape. Study champions (1 per practice) will support the PIVOT with MI strategy throughout the remainder of the intervention period by soliciting periodic feedback from providers on use of the intervention and addressing implementation issues with the study team.

C.6.g. Data Collection and Implementation: During the intervention period, all English- and Spanish-speaking parents of newborns will be screened with the PACV-SF prior to their initial visit at a participating practice by integrating the PACV-SF into standard practice workflow (see *C.6.e.1.*). The PACV-SF will also include demographic items that have been associated with under-immunization (birth order of their child, household income, marital status, parent self-designated race/ethnicity, gender, and number of children in their household)[31,113-115] and be embedded in a larger survey to minimize ascertainment bias.

To assess intervention fidelity (Aim 2a), a study RA will approach by phone English-speaking VHPs attending intervention practices who answer affirmatively to an item on the PACV-SF asking for their permission to contact them to determine their interest in participating in a study of provider-parent communication at health supervision visits in which their child's visit will be videotaped. We will describe the study generally to parents in order to minimize the chance that parents alter their behavior to meet observer expectations. We will approach VHPs continuously throughout the intervention period to obtain a collection of

videotaped encounters at different time periods to assess whether there is a waning of fidelity over time. We have chosen videotaping because it captures aspects of nonverbal communication that are (a) not captured by audiotaping alone and are essential to our study of vaccine acceptance (e.g. head nodding) and (b) necessary to provide visual feedback to providers during the 12 month refresher session (see *C.6.f.*).[116] For those VHPs who are interested (based on our past studies, we expect >70% will be interested [see *C.1.c.*]), the study RA will meet the parent at their child's next health supervision visit to obtain their written informed consent. The child's visit will then be videotaped using a video-recorder equipped with a wide-angle lens and positioned in a corner of the exam room near the ceiling, consistent with the methods we have used in preliminary studies (see *C.1.c.*). All videotaped visits will be transcribed. Parents will receive a $25 gift card for participating.

<u>C.6.h. Blinding:</u> Given our intervention, it is not possible to blind providers or investigators to study-arm allocation. However, our analysts will be blinded to study-arm allocation. We will also minimize selection bias by including all parents whose newborns receive health supervision at participating practices in the study and analysis. We will minimize participant bias by blinding providers to PACV-SF scores of parents and parent ascertainment bias by masking the PACV-SF during enrollment by embedding it in a larger survey.

<u>C.6.i. Primary Study Outcome:</u> **Our primary outcome will be the immunization status of children of VHPs at 19 months, 0 days of age** expressed as the mean percentage of days under-immunized from birth to 19 months for 22 doses of 8 vaccines recommended during this interval (3 Hepatitis B, 3 Rotavirus, 4 Diphtheria, tetanus, and acellular pertussis [DTaP], 3 *Haemophilus influenzae*, type b [Hib], 4 Pneumococcal conjugate, 3 Inactivated poliovirus [IPV], 1 Measles, mumps, rubella [MMR], and 1 Varicella). To calculate the mean percentage of days under-immunized, we will sum the days late for each dose and divide this by the maximum number of days a child could be late if they had received none of the total 22 doses for the 8 vaccines by 19 months. We have chosen to use the mean percentage of days under-immunized because it is a sensitive measure of under-immunization by accounting for missed vaccines doses and delay in receipt of vaccines.[117] We have successfully used this measure in previous studies (see *C.1.a.* and *C.1.c.*). We will obtain immunization data from WAIIS and CIIS as described in *C.6.e.4.* As an exploratory secondary outcome, we will also measure the immunization status of children of non-VHPs (expressed as the mean percentage of days under-immunized) since the PIVOT with MI intervention involves use of the presumptive format with all patients and therefore has the potential to also impact the vaccine behavior of non-VHPs.

<u>C.6.j. Analysis:</u> We will conduct an intention-to-treat analysis. The patient will be the unit of analysis. We will examine baseline characteristics of parents in control and intervention arms using Pearson's $\chi 2$ tests (or Fisher's exact tests) for categorical variables and t-tests for continuous variables to assess for any unbalanced confounders. Any unbalanced confounders will be controlled for as covariates in all subsequent regression analyses. We will also compare baseline characteristics between parents with and without missing data. We will apply sensitivity analysis and multiple imputations techniques to address missing data.

Given the nested structure of the data, we will apply mixed effects regression models to examine the effects of the intervention on our primary outcome while controlling for covariates and accounting for correlations due to clustering. Let y_{ijk} denote the percent days under-immunized for child k who receives care from provider j at clinic i, let T_{ijk} be the intervention indicator (0=control, 1=intervention) for the child, let $\boldsymbol{X}_{ijk}$ be the collection of covariates that includes potential confounders (at patient, provider, or practice levels), then a mixed model that accounts for this specific nesting data structure can be specified by

$$y_{ijk} = T_{jik}\alpha + \boldsymbol{X}_{ijk}\beta + Z^{(3)}_{ijk}\, u^{(3)}_{i} + Z^{(2)}_{ijk}\, u^{(2)}_{ij} + \epsilon_{ijk}, \qquad (1)$$

for $k = 1, \cdots, n_{ij}$ first-level observations (patient) nested within $j = 1, \cdots, M_i$ second level groups (provider), which are nested within $i = 1, \cdots, M$ third-level groups (practices). $Z^{(3)}_{ijk}$ and $Z^{(2)}_{ijk}$ are design matrices for the third- and second-level random effects. Further normality and independence assumptions will be placed on random effects and error terms if the outcome is continuous. The above model specification represents a multi-level linear mixed model. Parameter estimates will be obtained through restricted maximum likelihood estimation. Robust sandwich standard error estimates to account for the clustering will be used for inference. In the above model specification, the parameter α captures the expected difference between intervention and control arms and will be our focus of inference. For binary outcomes, the above model specification can be adapted to mixed effects logistic regression with similar estimation and inference techniques.[118]

For our fidelity study (Aim 2a), we will use conversation analysis (CA) to analyze use of the presumptive format to initiate the vaccine recommendation in videotaped data and the Motivational Interviewing Treatment Integrity (MITI) 4 system[119] to assess MI fidelity. CA is the same analytic technique we have used in our preliminary work (see *C.1.c.*) and searches for patterns in the provider-parent interaction in which a behavior is systematically used to accomplish a social action either vocally or nonvocally.[120] MITI utilizes a 5-point Likert type scale to assess four MI dimensions (cultivating change talk, softening sustain talk,

partnership, and empathy) and tallies 10 specific practitioner behaviors (questions, reflection, persuade with permission, giving information, affirm, emphasize autonomy, seek collaboration, persuade and confront). Summary scores include: (1) reflection to question ratio, (2) percentage of complex to simple reflections, and (3) percent of MI adherent statements.

Robinson will utilize a subset of the videotaped encounters to develop a coding scheme for the PIVOT with MI communication practices. Opel and O'Leary will teleconference weekly with Robinson during this analysis to review identified communication patterns and their correlation to the PIVOT with MI intervention. After completion of the coding scheme, Robinson will then train multiple coders from the study team on the coding scheme using 10% of the videotaped data. We will measure inter-rater reliability between the coders using up to an additional 20% of the data until κ scores reach a minimum of .70. Coders will then continue to independently code all remaining data (and recode the initial 10% of training data). All discrepancies in coding between the coders will be resolved through discussion with Robinson.

<u>C.6.k. Sample Sizes/Power Calculations:</u> Based on preliminary data (see *C.1.a.*), children of VHPs had a mean percentage of days under-immunized of 26.2% (standard deviation [SD] 29.8) from birth to 19 months of age for 6 vaccines combined (Hepatitis B, DTaP, Hib, IPV, MMR, and Varicella).[18] This corresponds to an average of 37 days late per vaccine dose. To be able to detect with adequate power (≥90%) a decrease in under-immunization of 10 days—a decrease that has the potential to be clinically meaningful since 27 days would not be considered late for most vaccines because of the 30 day window in which most vaccine doses are recommended[121]—we need to enroll 600 VHP/newborn pairs total, with 300 per arm and 25 at each of the 24 study practices (assuming an α of 0.05, a SD of 20, and an intraclass correlation coefficient for within-clinic correlation of 0.02[28]). Assuming a 10% prevalence of VHPs (see *C.1.b*),[28] we will need to approach 6,000 parents to reach our sample size goal. This is feasible to achieve in our designated 18 month recruitment period given our design to integrate PACV-SF screening of parents of newborns into standard practice workflow and our estimate (determined by spot chart reviews at PSPRN and COCONet practices) that there are ≥10 newborns per clinic per week in PSPRN and COCONet practices. If we vary our assumptions regarding VHP prevalence (7.5%) or SD (25), we would need to approach 8,000 and 12,000 parents, respectively, for adequate (≥90%) power to detect the same effect size, both of which remain feasible within the 18 month enrollment period assuming ≥10 newborns per clinic per week. If we have unbalanced enrollment across study arms (i.e. not 1:1 enrollment in intervention and control arms), we will continue enrollment beyond 600 VHP/newborn pairs total in order to reach at least 300 VHP/newborn pairs per arm given the impact of an imbalance on our power.

For our fidelity outcome (Aim 2a), power can be considered in terms of the number of videotaped interactions needed to develop this coding scheme. Based on our prior studies (see *C.1.c.*), 3 videotaped visits per provider should be adequate to achieve sufficient behavioral variation across providers. We will therefore videotape up to 360 encounters (up to 180 in each state), which averages to 3 videotaped encounters per practice provider.

<u>C.6.l. Strengths and Limitations:</u> We utilize a robust study design to minimize contamination as well as selection, ascertainment, and participant biases. Our use of 2 independent and well-established regional pediatric PBRNs facilitates generalizability and will provide a knowledge base for how an intervention can be integrated within a real-world practice setting, helping inform a dissemination and implementation follow-on study within the American Academy of Pediatrics' (AAP) PROS network and American Academy of Family Physicians' (AAFP) National Research Network (NRN). In measurement of the fidelity of the intervention, it is possible that provider behavior will be different when videotaped. However, other studies have demonstrated a negligible effect of the videotape on provider behavior.[122] By design, we only measure immunization status at 19 months of age given timeline constraints and therefore will not be able to assess the impact of the PIVOT with MI intervention on longer-term immunization status beyond 19 months of age.

C.6.m. <u>Potential Outcomes/Scientific Contribution:</u> If the PIVOT with MI intervention is effective in improving immunization status of children of VHPs, this study will address a critically important public health problem using an efficient and feasible program that contains scalable components to promote dissemination.

<u>C.7. Aim 3:</u> To evaluate the impact of the PIVOT with MI intervention on parent and provider experience.

<u>C.7.a. Overview and Design:</u> Given the preliminary association of a presumptive format for initiating the vaccine recommendation with *decreased* odds of a highly-rated visit experience (see *C.1.c.*), we will assess the PIVOT with MI intervention's impact on visit experience using a cross-sectional survey design of intervention and control VHPs. Also, given the preliminary association of decreased time spent discussing vaccines with VHPs and increased provider self-efficacy with use of MI in the PCOM intervention (see *C.1.d.*), we will use a pre-, interim -, and post - intervention survey of control and intervention providers to determine if the PIVOT

with MI intervention has a similar impact. Lastly, provider use of a presumptive initiation format and/or MI in vaccine discussions may increase across practices broadly over time given publication and dissemination of our preliminary work. Therefore, we are interested in assessing use of these techniques by both control and intervention providers at baseline and after the intervention to better interpret our study results.

C.7.b. Study Population: VHPs from both control and intervention practices who indicate on the embedded PACV-SF that study staff can contact them about participation in additional study activities will be eligible. All pediatric providers (MD, DO, ARNP, PA-C) from control and intervention practices will be eligible.

C.7.c. Data Collection and Implementation: Each videotaped encounter participant will receive a $25 gift card as a thank you for their participation.

We will administer a post-visit experience survey that has been adapted from the Outpatient Satisfaction Questionnaire[123] and the Satisfaction with Immunization Service Questionnaire[124] (Appendix 2) to VHPs in both control and intervention sites. Due to COVID implications, we will separate the post-visit experience survey from the in-person visit where originally it was to be administered immediately after one of their child's health supervision visits. For intervention and control VHPs, contact will be made after their child's 2-month visit if the parent gave their contact information on the PACV survey and agreed to contacted to potentially participate in future research. A RA will call and contact the parent to gauge interest in the survey and if so, will proceed with administering the survey either via an emailed link or over the phone, while the RA will enter their answers in real time in REDCap. Once the survey is completed, the parent will receive a $20 gift card as a thank you.

To assess provider time, communication approach, and self-efficacy in discussing vaccines with parents, we will administer surveys to all study providers at baseline, after the completion of the trainings in the intervention practices and at study completion. Baseline surveys will also include items querying provider demographic and practice characteristics. The survey instrument will be modified from a survey used in the PCOM project (see *C.1.d.*) and pilot tested (Appendix 3). The interim and post study surveys will have the same questions for both intervention and control providers, with a few additional questions evaluating the PIVOT with MI trainings and experiences among intervention providers only. Administration will be by paper or electronically, depending on practice preference. Participants will receive a $25 gift card for participation. In previous studies with similar methods, we've achieved 87-95% provider response rates.

An additional survey taken by each of our pediatric clinics will better help us understand the impact of COVID-19 on health supervision visits and vaccine delivery and uptake. This survey will be administered in the summer months of 2020.

C.7.d. Analysis: Since there is no optimal method for summarizing parent experience, we will use 2 dichotomization methods: the top-box method,[125-128] consistent with Consumer Assessment of Healthcare Providers and Systems survey scoring,[129] and an alternative method[123,130,131] in which an average score of ≥6 (out of 7) on each of the 15 items is indicative of a highly rated visit experience.[16] We will also summarize parent experience as a continuous variable. We will use Pearson's χ2 test to compare parent (binary) ratings of visit experience between control and intervention arms. We will apply mixed effects logistic regression model to account for within-clinic correlation and any unbalanced confounding factors. When summarizing parent experience as a continuous variable, we will apply Box-Cox transformation if it is highly skewed and linear mixed effect models similar to Equation (1) to examine differences between control and intervention arms.

For the provider outcomes, we will compare the pre- and post-intervention proportions of control and intervention providers who (1) spent <10 minutes vs. 10+ minutes discussing vaccines at a typical visit and during visits with parents who had vaccine concerns; (2) used the presumptive and/or MI techniques in vaccine discussions with families (always, frequently, sometimes, never); and (3) perceived they were able to influence parents' vaccine decisions (Appendix 3). We will use Pearson's χ2 test for an unadjusted analysis of the pre-post differences between control and intervention arms and multivariable logistic regression for adjusted analyses that control for potential confounding variables such as provider demographic and practice characteristics. Planned subgroup analyses include examining outcomes by practice type (public/private), %VHPs in the practice, and number of practice providers. Models will account for clustering of outcomes by practices where needed.

C.7.e. Power calculation: For the parent visit experience outcome, we will have 80% power to detect a 13 percentage point difference in the proportion of parents who rate their visit experience highly between control and PIVOT with MI practices if we enroll 312 VHPs (156 per arm) assuming a baseline proportion of 72% of parents who rate their visit experience highly[16] and equal distribution of participants in control and PIVOT with MI practices. For each of the provider outcomes, we will have 80% power to detect a 12 percentage point pre-post difference between control (1 percentage point pre-post difference) and intervention providers (13 percentage point pre-post difference), assuming 60 providers in each arm. Overall, this number of providers

per arm for these provider outcomes seems feasible since we anticipate the proposed study will involve >120 total providers (5-7 providers per practice).

<u>C.7.f. Strengths and Limitations:</u> We are only including VHPs who give us permission to contact them. There may be differential participation rates among VHPs in the control and intervention arms, resulting in unequal distribution and bias. Though we will account for this in our analysis, our results may nonetheless not be generalizable to parents and children with less access to health care and to parents less likely to participate.

C.8. Future Directions

We have designed the proposed study with dissemination in mind. Study materials will be developed so that they may be easily adapted to other settings, with particular focus on having the online video module available for use by others immediately (see *C.5.e.*). In addition, should this intervention prove effective, we intend to collaborate with 2 national PBRNs—the AAP PROS Network and the AAFP NRN—to test the intervention on a broader scale (*see letters of support from the directors of PROS and NRN*).

C.9. Study Timeline

**Project Task**	**Year 1**				**Year 2**				**Year 3**				**Year 4**				**Year 5**			
**Quarters**	**1**	**2**	**3**	**4**	**1**	**2**	**3**	**4**	**1**	**2**	**3**	**4**	**1**	**2**	**3**	**4**	**1**	**2**	**3**	**4**
1) Obtain all needed Institutional Review Board approvals																				
2) Recruit PSPRN and COCONet practices																				
3) Develop PIVOT with MI provider training program																				
4) Obtain MOC-4 approval for PIVOT with MI program																				
5) Data collection for covariates in balanced randomization																				
6) Baseline provider survey and PIVOT with MI training																				
7) Parent/newborn enrollment period																				
8) Videotaping for fidelity of intervention																				
9) Refresher PIVOT with MI trainings																				
10) Parent visit experience surveys																				
11) Provider interim-intervention survey																				
12) COVID-19 survey to all practices																				
13) Access WAIIS and CIIS vaccine records																				
14) Provider post-intervention survey																				
15) Data analysis																				
16) Writing and dissemination																				

BIBLIOGRAPHY AND REFERENCES CITED

1. Hill HA, Elam-Evans LD, Yankey D, Singleton JA, Dietz V. Vaccination Coverage Among Children Aged 19-35 Months - United States, 2015. *Mmwr*. Oct 07 2016;65(39):1065-1071. doi:10.15585/mmwr.mm6539a4
2. Seither R, Calhoun K, Mellerson J, et al. Vaccination Coverage Among Children in Kindergarten - United States, 2015-16 School Year. *Mmwr*. Oct 07 2016;65(39):1057-1064. doi:10.15585/mmwr.mm6539a3
3. Sciences AAoAa. *Public Trust in Vaccines: Defining a Research Agenda*. 2014.
4. Omer SB, Richards JL, Ward M, Bednarczyk RA. Vaccination policies and rates of exemption from immunization, 2005-2011. *The New England journal of medicine*. Sep 20 2012;367(12):1170-1. doi:10.1056/NEJMc1209037
5. Richards JL, Wagenaar BH, Van Otterloo J, et al. Nonmedical exemptions to immunization requirements in California: a 16-year longitudinal analysis of trends and associated community factors. *Vaccine*. Jun 24 2013;31(29):3009-13. doi:10.1016/j.vaccine.2013.04.053
6. Hough-Telford C, Kimberlin DW, Aban I, et al. Vaccine Delays, Refusals, and Patient Dismissals: A Survey of Pediatricians. *Pediatrics*. Sep 2016;138(3)doi:10.1542/peds.2016-2127
7. Kempe A, O'Leary ST, Kennedy A, et al. Physician response to parental requests to spread out the recommended vaccine schedule. *Pediatrics*. Apr 2015;135(4):666-77. doi:10.1542/peds.2014-3474
8. Atwell JE, Van Otterloo J, Zipprich J, et al. Nonmedical vaccine exemptions and pertussis in California, 2010. *Pediatrics*. Oct 2013;132(4):624-30. doi:10.1542/peds.2013-0878
9. Omer SB, Enger KS, Moulton LH, Halsey NA, Stokley S, Salmon DA. Geographic clustering of nonmedical exemptions to school immunization requirements and associations with geographic clustering of pertussis. *American journal of epidemiology*. Dec 15 2008;168(12):1389-96.

10. Sadaf A, Richards JL, Glanz J, Salmon DA, Omer SB. A systematic review of interventions for reducing parental vaccine refusal and vaccine hesitancy. *Vaccine*. Sep 13 2013;31(40):4293-304. doi:10.1016/j.vaccine.2013.07.013
11. Assessing the State of Vaccine Confidence in the United States: Recommendations from the National Vaccine Advisory Committee: Approved by the National Vaccine Advisory Committee on June 10, 2015. *Public Health Rep*. Nov-Dec 2015;130(6):573-95.
12. Freed GL, Clark SJ, Butchart AT, Singer DC, Davis MM. Sources and perceived credibility of vaccine-safety information for parents. *Pediatrics*. May 2011;127 Suppl 1:S107-12.
13. Gust DA, Darling N, Kennedy A, Schwartz B. Parents with doubts about vaccines: which vaccines and reasons why. *Pediatrics*. Oct 2008;122(4):718-25.
14. Opel DJ, Heritage J, Taylor JA, et al. The Architecture of Provider-Parent Vaccine Discussions at Health Supervision Visits. *Pediatrics*. 2013;132(6):1037-1046. doi:10.1542/peds.2013-2037
15. Fredrickson DD, Davis TC, Arnould CL, et al. Childhood immunization refusal: provider and parent perceptions. *Family medicine*. Jun 2004;36(6):431-9.
16. Opel DJ, Mangione-Smith R, Robinson JD, et al. The Influence of Provider Communication Behaviors on Parental Vaccine Acceptance and Visit Experience. *American journal of public health*. Oct 2015;105(10):1998-2004. doi:10.2105/AJPH.2014.302425
17. Opel DJ, Taylor JA, Mangione-Smith R, et al. Validity and reliability of a survey to identify vaccine-hesitant parents. *Vaccine*. Sep 2 2011;29(38):6598-605.
18. Opel DJ, Taylor JA, Zhou C, Catz S, Myaing M, Mangione-Smith R. The relationship between parent attitudes about childhood vaccines survey scores and future child immunization status: a validation study. *JAMA Pediatr*. Nov 1 2013;167(11):1065-71. doi:10.1001/jamapediatrics.2013.2483
19. A CDC Framework for Preventing Infectious Diseases: Sustaining the Essentials and Innovating for the Future (2011).
20. Phadke VK, Bednarczyk RA, Salmon DA, Omer SB. Association Between Vaccine Refusal and Vaccine-Preventable Diseases in the United States: A Review of Measles and Pertussis. *JAMA*. Mar 15 2016;315(11):1149-58. doi:10.1001/jama.2016.1353
21. Glanz JM, Newcomer SR, Narwaney KJ, et al. A population-based cohort study of undervaccination in 8 managed care organizations across the United States. *JAMA Pediatr*. Mar 1 2013;167(3):274-81. doi:10.1001/jamapediatrics.2013.5021558057 [pii]
22. Glanz JM, McClure DL, Magid DJ, Daley MF, France EK, Hambidge SJ. Parental refusal of varicella vaccination and the associated risk of varicella infection in children. *Archives of pediatrics & adolescent medicine*. Jan 2010;164(1):66-70. doi:10.1001/archpediatrics.2009.244
23. Glanz JM, McClure DL, O'Leary ST, et al. Parental decline of pneumococcal vaccination and risk of pneumococcal related disease in children. *Vaccine*. Jan 29 2011;29(5):994-9. doi:10.1016/j.vaccine.2010.11.085
24. Glanz JM, Narwaney KJ, Newcomer SR, et al. Association between undervaccination with diphtheria, tetanus toxoids, and acellular pertussis (DTaP) vaccine and risk of pertussis infection in children 3 to 36 months of age. *JAMA Pediatr*. Nov 2013;167(11):1060-4. doi:10.1001/jamapediatrics.2013.2353
25. Invasive Haemophilus influenzae Type B disease in five young children--Minnesota, 2008. *Mmwr*. Jan 30 2009;58(3):58-60.
26. Hammer LD, Curry ES, Harlor AD, et al. Increasing immunization coverage. *Pediatrics*. Jun 2010;125(6):1295-304. doi:10.1542/peds.2010-0743
27. Leask J. Target the fence-sitters. *Nature*. May 26 2011;473(7348):443-5. doi:10.1038/473443a
28. Henrikson NB, Opel DJ, Grothaus L, et al. Physician Communication Training and Parental Vaccine Hesitancy: A Randomized Trial. *Pediatrics*. Jul 2015;136(1):70-9. doi:10.1542/peds.2014-3199
29. Williams SE, Morgan A, Opel D, Edwards K, Weinberg S, Rothman R. Screening Tool Predicts Future Underimmunization Among a Pediatric Practice in Tennessee. *Clinical pediatrics*. Nov 18 2015;doi:10.1177/0009922815615823
30. Benin AL, Wisler-Scher DJ, Colson E, Shapiro ED, Holmboe ES. Qualitative analysis of mothers' decision-making about vaccines for infants: the importance of trust. *Pediatrics*. May 2006;117(5):1532-41.
31. Smith PJ, Chu SY, Barker LE. Children who have received no vaccines: who are they and where do they live? *Pediatrics*. Jul 2004;114(1):187-95.
32. Gust D, Brown C, Sheedy K, Hibbs B, Weaver D, Nowak G. Immunization attitudes and beliefs among parents: beyond a dichotomous perspective. *American journal of health behavior*. Jan-Feb 2005;29(1):81-92.

33. Kennedy AM, Brown CJ, Gust DA. Vaccine beliefs of parents who oppose compulsory vaccination. *Public Health Rep*. May-Jun 2005;120(3):252-8.
34. Gust DA, Kennedy A, Weber D, Evans G, Kong Y, Salmon D. Parents questioning immunization: evaluation of an intervention. *American journal of health behavior*. May-Jun 2009;33(3):287-98.
35. Omer SB, Salmon DA, Orenstein WA, deHart MP, Halsey N. Vaccine refusal, mandatory immunization, and the risks of vaccine-preventable diseases. *The New England journal of medicine*. May 7 2009;360(19):1981-8.
36. Smith PJ, Kennedy AM, Wooten K, Gust DA, Pickering LK. Association between health care providers' influence on parents who have concerns about vaccine safety and vaccination coverage. *Pediatrics*. Nov 2006;118(5):e1287-92.
37. McCauley MM, Kennedy A, Basket M, Sheedy K. Exploring the choice to refuse or delay vaccines: a national survey of parents of 6- through 23-month-olds. *Academic pediatrics*. Sep 2012;12(5):375-83. doi:10.1016/j.acap.2012.06.007
38. Daley MF, Crane LA, Chandramouli V, et al. Influenza among healthy young children: changes in parental attitudes and predictors of immunization during the 2003 to 2004 influenza season. *Pediatrics*. Feb 2006;117(2):e268-77.
39. Flood EM, Rousculp MD, Ryan KJ, et al. Parents' decision-making regarding vaccinating their children against influenza: A web-based survey. *Clinical therapeutics*. Aug 2010;32(8):1448-67. doi:10.1016/j.clinthera.2010.06.020
40. Gnanasekaran SK, Finkelstein JA, Hohman K, O'Brien M, Kruskal B, Lieu T. Parental perspectives on influenza vaccination among children with asthma. *Public Health Rep*. Mar-Apr 2006;121(2):181-8.
41. Nowalk MP, Lin CJ, Zimmerman RK, et al. Changes in parents' perceptions of infant influenza vaccination over two years. *Journal of the National Medical Association*. Jun 2007;99(6):636-41.
42. Brewer NT, Gottlieb SL, Reiter PL, et al. Longitudinal predictors of human papillomavirus vaccine initiation among adolescent girls in a high-risk geographic area. *Sexually transmitted diseases*. Mar 2011;38(3):197-204. doi:10.1097/OLQ.0b013e3181f12dbf
43. Guerry SL, De Rosa CJ, Markowitz LE, et al. Human papillomavirus vaccine initiation among adolescent girls in high-risk communities. *Vaccine*. Mar 09 2011;29(12):2235-41. doi:10.1016/j.vaccine.2011.01.052
44. Smith PJ, Stokley S, Bednarczyk RA, Orenstein WA, Omer SB. HPV vaccination coverage of teen girls: the influence of health care providers. *Vaccine*. Mar 18 2016;34(13):1604-10. doi:10.1016/j.vaccine.2016.01.061
45. Freeman VA, Freed GL. Parental knowledge, attitudes, and demand regarding a vaccine to prevent varicella. *American journal of preventive medicine*. Aug 1999;17(2):153-5.
46. Rosenthal SL, Kottenhahn RK, Biro FM, Succop PA. Hepatitis B vaccine acceptance among adolescents and their parents. *The Journal of adolescent health : official publication of the Society for Adolescent Medicine*. Oct 1995;17(4):248-54. doi:10.1016/1054-139X(95)00164-N
47. Ylitalo KR, Lee H, Mehta NK. Health care provider recommendation, human papillomavirus vaccination, and race/ethnicity in the US National Immunization Survey. *American journal of public health*. Jan 2013;103(1):164-9. doi:10.2105/AJPH.2011.300600
48. Brewer NT, Hall ME, Malo TL, Gilkey MB, Quinn B, Lathren C. Announcements Versus Conversations to Improve HPV Vaccination Coverage: A Randomized Trial. *Pediatrics*. Dec 05 2016;doi:10.1542/peds.2016-1764
49. Leask J, Kinnersley P, Willaby HW, Danchin M. Presumptive initiations in vaccine discussions with parents: acquiescence but at what cost? [E-letter]. Pediatrics; 2013. December 13, 2013. Accessed December 15, 2013. http://pediatrics.aappublications.org/content/132/6/1037/reply#pediatrics_el_56846
50. Kempe A, Daley MF, McCauley MM, et al. Prevalence of parental concerns about childhood vaccines: the experience of primary care physicians. *American journal of preventive medicine*. May 2011;40(5):548-55. doi:10.1016/j.amepre.2010.12.025
51. Miller WR. Motivational interviewing: research, practice, and puzzles. *Addictive behaviors*. Nov-Dec 1996;21(6):835-42.
52. Interviewing M. 01/02/2013, http://www.motivationalinterview.org/index.html
53. Berg CJ, Thomas JL, An LC, et al. Change in smoking, diet, and walking for exercise in Blacks. *Health Educ Behav*. Apr 2012;39(2):191-7. doi:10.1177/1090198111432252

54. Brand V, Bray K, Macneill S, Catley D, Williams K. Impact of single-session motivational interviewing on clinical outcomes following periodontal maintenance therapy. *International journal of dental hygiene*. Dec 24 2012;doi:10.1111/idh.12012
55. Cooper L. Combined motivational interviewing and cognitive-behavioral therapy with older adult drug and alcohol abusers. *Health & social work*. Aug 2012;37(3):173-9.
56. Leask J, Kinnersley P, Jackson C, Cheater F, Bedford HE, Rowles G. Communicating with parents about vaccination: a framework for health professionals. *BMC Pediatr*. Sep 21 2012;12(1):154. doi:10.1186/1471-2431-12-154
57. McCain J. To Heal the Body, Get Into the Patient's Head: Motivational Interviewing: To improve adherence. *Biotechnology healthcare*. Winter 2012;9(4):10-2.
58. Thomas ML, Elliott JE, Rao SM, Fahey KF, Paul SM, Miaskowski C. A randomized, clinical trial of education or motivational-interviewing-based coaching compared to usual care to improve cancer pain management. *Oncology nursing forum*. Jan 2012;39(1):39-49. doi:10.1188/12.onf.39-49
59. Boutin-Foster C, Scott E, Rodriguez A, et al. The Trial Using Motivational Interviewing and Positive Affect and Self-Affirmation in African-Americans with Hypertension (TRIUMPH): From Theory to Clinical Trial Implementation. *Contemporary clinical trials*. Feb 8 2013;doi:10.1016/j.cct.2013.02.002
60. Gabbay RA, Anel-Tiangco RM, Dellasega C, Mauger DT, Adelman A, Horn DH. Diabetes Nurse Case Management and Motivational Interviewing for Change (DYNAMIC): Results of a 2-year Randomized Controlled Pragmatic Trial. *Journal of diabetes*. Feb 1 2013;doi:10.1111/1753-0407.12030
61. Gance-Cleveland B. Motivational interviewing for adolescent obesity. *The American journal of nursing*. Jan 2013;113(1):11. doi:10.1097/01.NAJ.0000425726.07495.ff
62. Hides L, Carroll S, Scott R, Cotton S, Baker A, Lubman DI. Quik fix: a randomized controlled trial of an enhanced brief motivational interviewing intervention for alcohol/cannabis and psychological distress in young people. *Psychotherapy and psychosomatics*. 2013;82(2):122-4. doi:10.1159/000341921
63. Klimas J, Field CA, Cullen W, et al. Psychosocial interventions to reduce alcohol consumption in concurrent problem alcohol and illicit drug users: Cochrane Reviewa. *Systematic reviews*. 2013;2(1):3. doi:10.1186/2046-4053-2-3
64. Nirenberg T, Baird J, Longabaugh R, Mello MJ. Motivational counseling reduces future police charges in court referred youth. *Accident; analysis and prevention*. Jan 16 2013;53C:89-99. doi:10.1016/j.aap.2013.01.006
65. Rongkavilit C, Naar-King S, Wang B, et al. Motivational Interviewing Targeting Risk Behaviors for Youth Living with HIV in Thailand. *AIDS and behavior*. Jan 17 2013;doi:10.1007/s10461-013-0407-2
66. Schoonheim-Klein M, Gresnigt C, van der Velden U. Influence of dental education in motivational interviewing on the efficacy of interventions for smoking cessation. *European journal of dental education : official journal of the Association for Dental Education in Europe*. Feb 2013;17(1):e28-33. doi:10.1111/j.1600-0579.2012.00755.x
67. Ski CF, Thompson DR. Motivational interviewing as a brief intervention to improve cardiovascular health. *European journal of cardiovascular nursing : journal of the Working Group on Cardiovascular Nursing of the European Society of Cardiology*. Jan 9 2013;doi:10.1177/1474515112472271
68. Tse MM, Vong SK, Tang SK. Motivational interviewing and exercise programme for community-dwelling older persons with chronic pain: a randomised controlled study. *Journal of clinical nursing*. Jan 2 2013;doi:10.1111/j.1365-2702.2012.04317.x
69. Fierman AH. Voices from the field: controversies in vaccine mandates. *Current problems in pediatric and adolescent health care*. Mar 2010;40(3):59.
70. Opel DJ, Feemster KA, Omer SB, Orenstein WA, Richter M, Lantos JD. A 6-month-old with vaccine-hesitant parents. *Pediatrics*. Mar 2014;133(3):526-30. doi:10.1542/peds.2013-2723
71. Bloom BR, Marcuse E, Mnookin S. Addressing vaccine hesitancy. *Science*. Apr 25 2014;344(6182):339. doi:10.1126/science.1254834
72. Volpp KG, Asch DA. Make the Healthy Choice the Easy Choice: Using Behavioral Economics to Advance a Culture of Health. *QJM : monthly journal of the Association of Physicians*. Nov 01 2016;doi:10.1093/qjmed/hcw190
73. Buttenheim AM, Asch DA. Leveraging Behavioral Insights to Promote Vaccine Acceptance: One Year After Disneyland. *JAMA Pediatr*. Jul 1 2016;170(7):635-6. doi:10.1001/jamapediatrics.2016.0192
74. Ariely D. Predictably Irrational. New York, NY: Harper-Collins; 2009.
75. Johnson EJ, Goldstein D. Medicine. Do defaults save lives? *Science*. Nov 21 2003;302(5649):1338-9. doi:10.1126/science.1091721

76. Bazerman MH, Moore DA. *Judgment in Managerial Decision-Making*. Wiley; 2013.
77. Knapp M. *Handbook of Interpersonal Communication, 3rd Edition*. Sage Publishing; 2002.
78. Simis MJ, Madden H, Cacciatore MA, Yeo SK. The lure of rationality: Why does the deficit model persist in science communication? *Public Underst Sci*. May 2016;25(4):400-14. doi:10.1177/0963662516629749
79. Nyhan B, Reifler J. Does correcting myths about the flu vaccine work? An experimental evaluation of the effects of corrective information. *Vaccine*. Jan 09 2015;33(3):459-64. doi:10.1016/j.vaccine.2014.11.017
80. Nyhan B, Reifler J, Richey S, Freed GL. Effective messages in vaccine promotion: a randomized trial. *Pediatrics*. Apr 2014;133(4):e835-42. doi:10.1542/peds.2013-2365
81. Kempe A, Albright K, O'Leary S, et al. Effectiveness of primary care-public health collaborations in the delivery of influenza vaccine: a cluster-randomized pragmatic trial. *Preventive medicine*. Dec 2014;69:110-6. doi:10.1016/j.ypmed.2014.08.019
82. O'Leary ST, Lee M, Lockhart S, et al. Effectiveness and Cost of Bidirectional Text Messaging for Adolescent Vaccines and Well Care. *Pediatrics*. Nov 2015;136(5):e1220-7. doi:10.1542/peds.2015-1089
83. Payne DC, Humiston S, Opel D, et al. A multi-center, qualitative assessment of pediatrician and maternal perspectives on rotavirus vaccines and the detection of Porcine circovirus. *BMC pediatrics*. Sep 26 2011;11:83. doi:10.1186/1471-2431-11-83
84. Opel DJ, Mangione-Smith R, Taylor JA, et al. Development of a survey to identify vaccine-hesitant parents: The parent attitudes about childhood vaccines survey. *Hum Vaccin*. Apr 1 2011;7(4):419-425.
85. MacDonald S, Dube E, Gagnon D, et al. Validating a 'Vaccine Hesitancy' instrument in a cohort of Alberta parents: A Canadian Immunization Research Network (CIRN) study. presented at: Canadian Immnization Conference; 2016; Ottawa, Ontario, Canada.
86. Williams SE, Morgan A, Opel D, Edwards K, Weinberg S, Rothman R. Screening Tool Predicts Future Underimmunization Among a Pediatric Practice in Tennessee. *Clinical pediatrics*. Jun 2016;55(6):537-42. doi:10.1177/0009922815615823
87. Strelitz B, Gritton J, Klein EJ, et al. Parental vaccine hesitancy and acceptance of seasonal influenza vaccine in the pediatric emergency department. *Vaccine*. Apr 8 2015;33(15):1802-7. doi:10.1016/j.vaccine.2015.02.034
88. Organization WH. Process of translation and adaptation of instruments. Accessed January 22, 2017. http://www.who.int/substance_abuse/research_tools/translation/en/
89. Opel DJ, Robinson JD, Heritage J, Korfiatis C, Taylor JA, Mangione-Smith R. Characterizing providers' immunization communication practices during health supervision visits with vaccine-hesitant parents: A pilot study. *Vaccine*. Feb 8 2012;30(7):1269-75.
90. Resnicow K, Teixeira PJ, Williams GC. Efficient Allocation of Public Health and Behavior Change Resources: The "Difficulty by Motivation" Matrix. *American journal of public health*. Jan 2017;107(1):55-57. doi:10.2105/AJPH.2016.303526
91. Davis DA, Thomson MA, Oxman AD, Haynes RB. Changing physician performance. A systematic review of the effect of continuing medical education strategies. *JAMA*. Sep 06 1995;274(9):700-5.
92. Grimshaw JM, Shirran L, Thomas R, et al. Changing provider behavior: an overview of systematic reviews of interventions. *Medical care*. Aug 2001;39(8 Suppl 2):II2-45.
93. Bauchner H, Simpson L, Chessare J. Changing physician behaviour. *Archives of disease in childhood*. Jun 2001;84(6):459-62.
94. Merriam SB, Cafrrarella RS. *Learing in Adulthood*. Jossey-Bass; 2008.
95. Bero LA, Grilli R, Grimshaw JM, Harvey E, Oxman AD, Thomson MA. Closing the gap between research and practice: an overview of systematic reviews of interventions to promote the implementation of research findings. The Cochrane Effective Practice and Organization of Care Review Group. *BMJ (Clinical research ed*. Aug 15 1998;317(7156):465-8.
96. Grimshaw JM, Eccles MP, Walker AE, Thomas RE. Changing physicians' behavior: what works and thoughts on getting more things to work. *The Journal of continuing education in the health professions*. Fall 2002;22(4):237-43.
97. O'Brien MA, Rogers S, Jamtvedt G, et al. Educational outreach visits: effects on professional practice and health care outcomes. *Cochrane database of systematic reviews*. 2007;(4):CD000409. doi:10.1002/14651858.CD000409.pub2
98. Rao JK, Anderson LA, Inui TS, Frankel RM. Communication interventions make a difference in conversations between physicians and patients: a systematic review of the evidence. *Medical care*. Apr 2007;45(4):340-9.

99. Ivers N, Jamtvedt G, Flottorp S, et al. Audit and feedback: effects on professional practice and healthcare outcomes. *Cochrane database of systematic reviews*. Jun 13 2012;(6):CD000259. doi:10.1002/14651858.CD000259.pub3
100. Dearing JW. Applying Diffusion of Innovation Theory to Intervention Development. *Res Soc Work Pract*. Sep 01 2009;19(5):503-518. doi:10.1177/1049731509335569
101. Cook DA, Blachman MJ, West CP, Wittich CM. Physician Attitudes About Maintenance of Certification: A Cross-Specialty National Survey. *Mayo Clin Proc*. Oct 2016;91(10):1336-1345. doi:10.1016/j.mayocp.2016.07.004
102. Fiks AG, Luan X, Mayne SL. Improving HPV Vaccination Rates Using Maintenance-of-Certification Requirements. *Pediatrics*. Mar 2016;137(3):e20150675. doi:10.1542/peds.2015-0675
103. Huang JS, Chun S, Sandhu A, Terrones L. Quality improvement in childhood obesity management through the maintenance of certification process. *J Pediatr*. Nov 2013;163(5):1313-6 e1. doi:10.1016/j.jpeds.2013.05.011
104. Glynn RJ, Brookhart MA, Stedman M, Avorn J, Solomon DH. Design of cluster-randomized trials of quality improvement interventions aimed at medical care providers. *Med Care*. Oct 2007;45(10 Supl 2):S38-43. doi:10.1097/MLR.0b013e318070c0a0
105. Raab GM, Butcher I. Balance in cluster randomized trials. *Stat Med*. Feb 15 2001;20(3):351-65.
106. Kraschnewski JL, Keyserling TC, Bangdiwala SI, et al. Optimized probability sampling of study sites to improve generalizability in a multisite intervention trial. *Prev Chronic Dis*. Jan 2010;7(1):A10.
107. Chaudhary MA, Moulton LH. A SAS macro for constrained randomization of group-randomized designs. *Comput Methods Programs Biomed*. Sep 2006;83(3):205-10. doi:10.1016/j.cmpb.2006.04.011
108. Dickinson LM, Beaty B, Fox C, et al. Pragmatic Cluster Randomized Trials Using Covariate Constrained Randomization: A Method for Practice-based Research Networks (PBRNs). *Journal of the American Board of Family Medicine : JABFM*. Sep-Oct 2015;28(5):663-72. doi:10.3122/jabfm.2015.05.150001
109. Kempe A, O'Leary ST, Shoup JA, et al. Parental Choice of Recall Method for HPV Vaccination: A Pragmatic Trial. *Pediatrics*. Mar 2016;137(3):e20152857. doi:10.1542/peds.2015-2857
110. O'Leary S, Pyrzanowski J, Brewer S, et al. A Pragmatic Cluster-Randomized Trial to Increase Uptake of Vaccines During Pregnancy. *Open Forum Infectious Diseases*. 2016;3(suppl_1):733-733. doi:10.1093/ofid/ofw172.596
111. Centers for Disease C, Prevention. Progress in immunization information systems - United States, 2012. *Mmwr*. Dec 13 2013;62(49):1005-8.
112. Jackson ML, Henrikson NB, Grossman DC. Evaluating Washington State's immunization information system as a research tool. *Academic pediatrics*. Jan-Feb 2014;14(1):71-6. doi:10.1016/j.acap.2013.10.002
113. Smith PJ, Humiston SG, Parnell T, Vannice KS, Salmon DA. The association between intentional delay of vaccine administration and timely childhood vaccination coverage. *Public Health Rep*. Jul-Aug 2010;125(4):534-41.
114. Smith PJ, Humiston SG, Marcuse EK, et al. Parental delay or refusal of vaccine doses, childhood vaccination coverage at 24 months of age, and the Health Belief Model. *Public Health Rep*. Jul-Aug 2011;126 Suppl 2:135-46.
115. Gust DA, Strine TW, Maurice E, et al. Underimmunization among children: effects of vaccine safety concerns on immunization status. *Pediatrics*. Jul 2004;114(1):e16-22.
116. Waitzkin H. On studying the discourse of medical encounters. A critique of quantitative and qualitative methods and a proposal for reasonable compromise. *Medical care*. Jun 1990;28(6):473-88.
117. Luman ET, Barker LE, Shaw KM, McCauley MM, Buehler JW, Pickering LK. Timeliness of childhood vaccinations in the United States: days undervaccinated and number of vaccines delayed. *Jama*. Mar 9 2005;293(10):1204-11.
118. McCullough CE, Searle SR, Neuhaus JM. *Generalized, Linear, and Mixed Models*. 2nd ed. John Wiley & Sons; 2011.
119. Moyers TB, Rowell LN, Manuel JK, Ernst D, Houck JM. The Motivational Interviewing Treatment Integrity Code (MITI 4): Rationale, Preliminary Reliability and Validity. *Journal of substance abuse treatment*. Jun 2016;65:36-42. doi:10.1016/j.jsat.2016.01.001
120. Heritage J, Maynard DW. *Communication in medical care : interaction between primary care physicians and patients*. [1st ed. Studies in interactional sociolinguistics ; 20. Cambridge University Pres; 2006:xix, 488 p.
121. Committee On Infectious D. Recommended childhood and adolescent immunization schedule--United States, 2014. *Pediatrics*. Feb 2014;133(2):357-63. doi:10.1542/peds.2013-3965

122. Themessl-Huber M, Humphris G, Dowell J, Macgillivray S, Rushmer R, Williams B. Audio-visual recording of patient-GP consultations for research purposes: a literature review on recruiting rates and strategies. *Patient education and counseling*. May 2008;71(2):157-68. doi:10.1016/j.pec.2008.01.015

123. Hays RD. *The Outpatient Satisfaction Questionnaire (OSQ-37): Executive Summary*. RAND; 1995.

124. Tickner S, Leman PJ, Woodcock A. Design and validation of the Satisfaction With Immunisation Service Questionnaire (SWISQ). *Vaccine*. Aug 16 2010;28(36):5883-90. doi:10.1016/j.vaccine.2010.06.055

125. Rubin HR, Gandek B, Rogers WH, Kosinski M, McHorney CA, Ware JE, Jr. Patients' ratings of outpatient visits in different practice settings. Results from the Medical Outcomes Study. *JAMA*. Aug 18 1993;270(7):835-40.

126. Wissow LS, Roter D, Bauman LJ, et al. Patient-provider communication during the emergency department care of children with asthma. The National Cooperative Inner-City Asthma Study, National Institute of Allergy and Infectious Diseases, NIH, Bethesda, MD. *Medical care*. Oct 1998;36(10):1439-50.

127. Bean-Mayberry BA, Chang CC, McNeil MA, Whittle J, Hayes PM, Scholle SH. Patient satisfaction in women's clinics versus traditional primary care clinics in the Veterans Administration. *Journal of general internal medicine*. Mar 2003;18(3):175-81.

128. Tom JO, Mangione-Smith R, Solomon C, Grossman DC. Integrated personal health record use: association with parent-reported care experiences. *Pediatrics*. Jul 2012;130(1):e183-90. doi:10.1542/peds.2011-1786

129. Foundation AIoRobotRWJ. How to Report Results of the CAHPS Clinician & Group Survey. Accessed November 7, 2013. http://www.rwjf.org/en/research-publications/find-rwjf-research/2010/09/how-to-report-results-of-the-cahps-clinician---group-survey.html

130. Stewart MA. What is a successful doctor-patient interview? A study of interactions and outcomes. *Social science & medicine (1982)*. 1984;19(2):167-75.

131. Yancy WS, Jr., Macpherson DS, Hanusa BH, et al. Patient satisfaction in resident and attending ambulatory care clinics. *Journal of general internal medicine*. Nov 2001;16(11):755-62.
