# Supplementary material for: Tiered Clinician Vaccine Communication Strategy to Improve Childhood Vaccine Uptake: A Cluster Randomized Clinical Trial
Source: JAMA Netw Open. 2025 Apr 30;8(4):e257814. doi: 10.1001/jamanetworkopen.2025.7814 (PMC12044505; doi:10.1001/jamanetworkopen.2025.7814)
Supplement: Supplement 2. — eTable 1. The Short Form of the Parent Attitudes about Childhood Vaccines (PACV) Survey eTable 2. Vaccine Codes Used for Analysis eTable 3. Specific Parameters for Determining Days Under-Immunized at 19 Months, 0 Days eTable 4. Results of Covariate-Constrained Randomization eAppendix. Training Curriculum for Clinicians (MD/DO, ARNP, PA-C) at Intervention Clinics eReferences [file jamanetwopen-e257814-s002.pdf]

Supplemental Online Content

Opel DJ, Robinson JD, Zhou C, et al. Tiered clinician vaccine communication strategy to improve childhood vaccine uptake: a cluster randomized clinical trial. *JAMA Netw Open*. Published online April 30, 2025. doi:10.1001/jamanetworkopen.2025.7814

eTable 1. The Short Form of the Parent Attitudes about Childhood Vaccines (PACV) Survey

eTable 2. Vaccine Codes Used for Analysis

eTable 3. Specific Parameters for Determining Days Under-Immunized at 19 Months, 0 Days

eTable 4. Results of Covariate-Constrained Randomization

eAppendix. Training Curriculum for Clinicians (MD/DO, ARNP, PA-C) at Intervention Clinics

eReferences

This supplemental material has been provided by the authors to give readers additional information about their work.

© 2025 Opel DJ et al. *JAMA Netw Open.*

eTable 1. The Short Form of the Parent Attitudes about Childhood Vaccines (PACV) Survey

No.	Item	Hesitant Response
1	Have you ever delayed having your child get a shot for reasons other than illness or allergy?*	Yes
2	How concerned are you that a shot might not prevent the disease?	Very concerned, somewhat concerned, or not sure
3	Overall, how hesitant about childhood shots would you consider yourself to be?	Very hesitant, somewhat hesitant, or not sure
4	I trust the information I receive about shots.	Strongly disagree, disagree, or not sure
*Only delay of the birth dose of the hepatitis B vaccine would be relevant for parents of children ≤2 months old, as was the case in the PACV validation study[1]		

eTable 2. Vaccine Codes Used for Analysis

Short Description	WAIIS numeric code	CVX code
MMR	3	3
Hep B, adolescent or pediatric/Hep B Ped/Adol - Preserv Free[a]	8 / 100	8
IPV	10	10
Hib, unspecified formulation	17	17
DTaP	20	20
varicella	21	21
Hib (PRP-OMP)	23	49
Hib (PRP-T)	24	48
DT (pediatric)	28	28
Hep B, unspecified formulation	45	45
MMRV	54	94
rotavirus, pentavalent	116	116
rotavirus, monovalent	119	119
DTaP-Hep B-IPV	203	110
polio, unspecified formulation	204	89
pneumococcal, unspecified formulation	205	109
DTaP, 5 pertussis antigens	210	106
DTaP, unspecified formulation	600	107
rotavirus, unspecified formulation	608	122
Pneumococcal conjugate PCV 13	614	133
DTaP-Hib-IPV	920	120
DTaP-IPV[b]	974	130
Pneumococcal conjugate, unspecified formulation	995	152
DTaP,IPV,Hib,HepB	2034	146
Pneumococcal conjugate PCV15, polysaccharide CRM197 conjugate, adjuvant, PF[c]	3019	215

Footnotes: CVX codes are vaccine administered codes developed and maintained by the Center for Disease Control and Prevention's (CDC) National Center of Immunization and Respiratory Diseases and utilized by the Colorado Immunization Information System (CIIS). The Washington Immunization Information System (WAIIS) maintains a separate numeric code system with some overlap with CVX codes. Unless noted below, vaccine codes were excluded if they were 1) not part of the pre-specified list of vaccines included in our PIVOT primary outcome; 2) not indicated for children at the age of the PIVOT sample (0-19 months); 3) were not active vaccines per the CDC CVX code database; or 4) were non-US vaccines per the CDC CVX code database.

[a]These two Hep B vaccines have separate WAIIS vaccine codes but ultimately are both converted to CVX code 08; therefore, the WA site used both WAISS vaccine codes and the CO site used the one CVX code that corresponds to both these vaccine types.

[b]Indicated only for age ≥4 years, and therefore outside of the study sample age range of birth to 19 months; however, this vaccine was still included since DTaP-IPV doses erroneously given to children <4 would be counted as valid for DTaP doses 1-4 or IPV doses 1-3 per the CDC.
[c]The Food and Drug Administration licensed PCV15 on June 22, 2022 for use in children 6 weeks through 17 years of age. Since the last enrolled study participant did not turn 19 months until March 26, 2023, conceivably some PIVOT participants could have received this vaccine, so it was included.

© 2025 Opel DJ et al. *JAMA Netw Open.*

eTable 3. Specific Parameters for Determining Days Under-Immunized at 19 months, 0 days[#]

Vaccination	Recommended age (month)	Dose #	Age in days when under-immunization count initiated	Minimum acceptable age	Minimum interval between doses
**Hepatitis B (HepB)**	Birth	1	32	Birth	-
	1-2	2	93	4 weeks	4 weeks (28d)
	6-18	3	580	24 weeks	8 weeks (56d)
			Max Days: 580 (19 months, 0 days) – 32 = 548		
**Rotavirus (RV)**	2	1	93	6 weeks	-
	4	2	154	10 weeks	4 weeks
	6[^]	3	215	14 weeks	4 weeks
			Max Days: 245 (8 months, 0 days, as RV is not indicated beyond this age) - 93 = 152		
**Diphtheria and tetanus toxoids and acellular pertussis (DTaP)**	2	1	93	6 weeks	-
	4	2	154	10 weeks	4 weeks
	6	3	215	14 weeks	4 weeks
	15-18	4	580[@]	12 months	6 months
			Max Days: 580 – 93 = 487		
**Haemophilus influenzae type b (Hib)**	2	1	93	6 weeks	-
	4	2	154	10 weeks	4 weeks
	6[*]	3	215	14 weeks	4 weeks
	12-15	4	489	12 months	8 weeks
			Max Days: 580 – 93 = 487		
**Pneumococcal conjugate (PCV)**	2	1	93	6 weeks	-
	4	2	154	10 weeks	4 weeks
	6	3	215	14 weeks	4 weeks
	12-15	4	489	12 months	8 weeks
			Max Days: 580 - 93 = 487		
**Poliovirus (IPV)**	2	1	93	6 weeks	-
	4	2	154	10 weeks	4 weeks
	6-18	3	580[@]	14 weeks	4 weeks
			Max Days: 580 – 93 = 487		
**Measles, mumps, rubella (MMR)**	12-15	1	489	12 months	-
			Max Days: 580 – 489 = 91		
**Varicella**	12-15	1	489	12 months	-
			Max Days: 580 – 489 = 91		
**TOTAL**		**23**	Total Max Days: 2830		

Footnotes:
[#]modified from previous similar analyses[1,2]

© 2025 Opel DJ et al. *JAMA Netw Open.*

^The 6 month dose of RV is not required if a child received Rotarix at 2 and 4 months. The 3-dose series at 2, 4 and 6 months only applies to those receiving RotaTeq. If any dose in the series was either RotaTeq or unknown, we defaulted to the 3-dose series.
*This 6 month dose of Hib is not required if a child received PedvaxHIB (Hib) at 2 and 4 months. The 4-dose series at 2, 4, 6 and 12-15 months only applies to those receiving ActHIB, Hiberix, or Pentacel.
@A child could not be considered late for the 4th dose of DTaP or the 3rd dose of IPV in our calculation since the first day a child could be considered late for each dose (given each are recommended through 18 months of age) was at the same age that we began counting days late (580 days, or 19 months, 0 days).

© 2025 Opel DJ et al. *JAMA Netw Open.*

eTable 4. Results of Covariate-Constrained Randomization

Variable	Intervention clinics (N=12)	Control clinics (N=12)
Percentage of parents with negative vaccine attitudes; mean, range	13% (0%, 30%)	12% (0%, 23%)
No. providers; mean, range	11 (2, 24)	7.8 (3, 12)
Percentage of VFC-eligible patients; mean, range	27% (3%, 79%)	24% (4%, 85%)

© 2025 Opel DJ et al. *JAMA Netw Open.*

eAppendix. Training Curriculum for Clinicians (MD/DO, ARNP, PA-C) at Intervention Clinics

The curriculum contained five components: (1) a brief, web-based video module to introduce the strategy; (2) one 60 minute baseline in-person training; (3) two 30-60 minute in-person or virtual refresher trainings 3-6 months and 9-12 months after the baseline training; (4) reference sheets of the main components of the strategy; and (5) a clinic champion who liaised with the study team at regular intervals to aid implementation. Recorded versions of the baseline and refresher sessions were made available to clinicians who were unable to attend. For the initial baseline training, 93% of clinicians (MD/DO, ARNP, PA-C) at intervention clinics participated; 82% and 77% clinicians participated in the two subsequent refresher trainings, respectively. Clinicians who completed the curriculum received Part 4 Maintenance of Certification credits.

eReferences

1. Opel DJ, Taylor JA, Zhou C, Catz S, Myaing M, Mangione-Smith R. The relationship between parent attitudes about childhood vaccines survey scores and future child immunization status: a validation study. *JAMA Pediatr*. Nov 1 2013;167(11):1065-71.
2. Luman ET, Barker LE, Shaw KM, McCauley MM, Buehler JW, Pickering LK. Timeliness of childhood vaccinations in the United States: days undervaccinated and number of vaccines delayed. *JAMA* Mar 9 2005;293(10):1204-11.

© 2025 Opel DJ et al. *JAMA Netw Open.*
