# Supplementary material for: Tiered Clinician Vaccine Communication Strategy to Improve Childhood Vaccine Uptake: A Cluster Randomized Clinical Trial
Source: JAMA Netw Open. 2025 Apr 30;8(4):e257814. doi: 10.1001/jamanetworkopen.2025.7814 (PMC12044505; doi:10.1001/jamanetworkopen.2025.7814)
Supplement: Supplement 3. — Data Sharing Statement [file jamanetwopen-e257814-s003.pdf]

Data Sharing Statement

Opel. Tiered Clinician Vaccine Communication Strategy to Improve Childhood Vaccine Uptake. *JAMA Netw Open*. Published April 30, 2025. doi:10.1001/jamanetworkopen.2025.7814

Data
Additional Information: ClinicalTrials.gov ID: NCT03885232; https://clinicaltrials.gov/study/NCT03885232
Data available: No
